# Biological Functional Class Enrichment Analysis with R, an Annotated Tutorial for Bench Scientists

**DOI:** 10.3390/mps9010028

**Published:** 2026-02-19

**Authors:** Kejin Hu

**Affiliations:** Department of Microbiology, Immunology and Genetics, College of Biomedical and Translational Sciences, University of North Texas Health Science Center, Fort Worth, TX 76107, USA; kejin.hu@unthsc.edu

**Keywords:** functional class enrichment analysis (FunCEA), gene ontology (GO), Kyoto Encyclopedia of Genes and Genomes (KEGG), *clusterProfiler*, pathway enrichment analysis, enrichment visualization, gene set enrichment analysis (GSEA), over-representation analysis (ORA), functional class scoring (FCS), category network plot (cnetplot)

## Abstract

High-throughput sequencing generally results in a list of genes. Which functional groups of genes among the DEGs are meaningful underlying factors to the differential biological/biomedical conditions under investigation? The process to find answers to this question can be called biological functional class enrichment analysis (FunCEA). R is a robust platform for FunCEA due to its accessibility by general users and availability of well-developed R packages for enrichment analysis and visualization, as well as for knowledge databases. Bench scientists in biomedical sciences need accessible and easy-to-understand protocols for FunCEA. This R tutorial provides detailed R scripts or command lines for FunCEA, as well as for data processing and visualization of the enrichment results. It keeps bench scientists in mind and provides supportive and apprehensible descriptions of the R scripts for each task (enrichment analysis, enrichment data processing, and visualization). It describes detailed procedures for the two popular FunCEA methods, the so-called over-representation analysis (ORA) and functional class scoring (FCS). The introduced FunCEA here uses three basic knowledge databases: gene ontology (GO), Kyoto Encyclopedia of Genes and Genomes (KEGG), and reactome. R codes for various visualizations (dot plot, term-gene network plot, enrichment map plot, ridge plot, and GSEA plot) are presented and annotated. Since all analyses are conducted in R, no commercial software is needed, yet *clusterProfiler* can directly access the latest KEGG knowledge database.

## 1. Introduction

**Figure 1:**
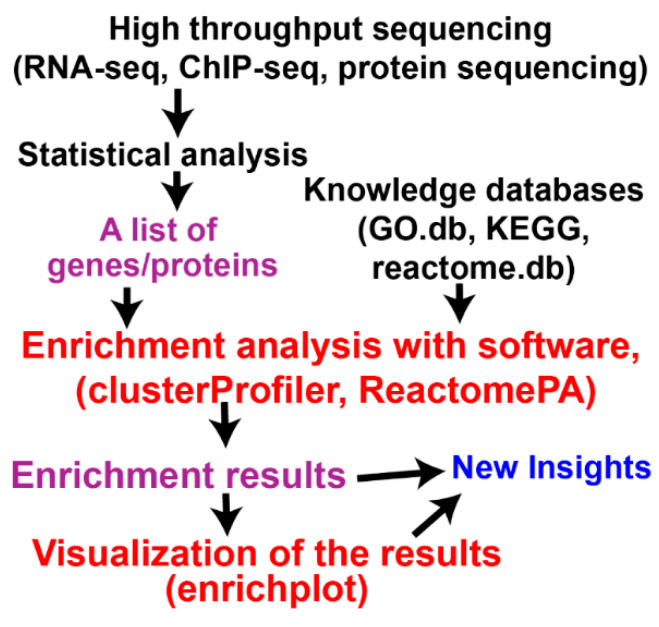
Overview of FunCEA. Those inside parentheses are R tools/resources used in this tutorial. Red indicates major computation steps in this tutorial; purple is computational results from previous steps as input data for the next step. Final outcome of the analysis is in blue.

High-throughput sequencing (HTS) is a commonplace approach in biological and biomedical sciences. HTS generally results in a list of genes/proteins that significantly differ between two conditions (Figure 1), which constitutes the secondary data in question. Accurate characterization of the resulting gene list is the essential part of HTS experiments. The list could include hundreds to thousands of genes. Functional characterization of that many genes is challenging, and designated software programs are needed to achieve efficient, quick, and accurate profiling of genes in the list. This process can be generally called biological functional class enrichment analysis (FunCEA for easy pronunciation). FunCEA tests whether each set of genes in the same functional group is significantly overrepresented in the resulting experimental gene list. FunCEA currently employs three different algorithms: over-representation analysis (ORA) [1], functional class scoring (FCS) [2], and pathway topology (PT) [3]. To functionally profile a list of experimental gene products, FunCEA requires gene function knowledge databases. There are many gene function knowledge databases, and this tutorial uses gene ontology (GO) annotation database (*GO.db*) [4], Kyoto Encyclopedia of Genes and Genomes (KEGG) [5], and reactome pathway database [6]. There are many R software packages for this purpose as well, and this tutorial introduces *clusterProfiler* [7,8] and *ReactomePA* [9]. The *clusterProfiler* package is used because it integrates two of the three approaches currently available, i.e., ORA and FCS. It also has several companion packages including *enrichplot* [10] and *ReactomePA*. Those R packages are widely used and well-maintained by the Yu group [7,11]. Visualization of enrichment results is critical in understanding and communication of the enrichment results. The *enrichplot* R package provides many functions to graphically summarize the enrichment results. The *enrichplot* package is based on the popular *ggplot2* graphics package, which can enhance the enrichment result visualization.

There are some web-based tools for FunCEA such as PANTHER (https://archive.pantherdb.org/, accessed on 17 January 2026) and DAVID (https://davidbioinformatics.nih.gov/, accessed on 17 January 2026), but the R-based methods using designated packages are more robust, versatile, and professional. Figure 1 depicts the FunCEA workflow. We will focus on ORA and FCS using the annotation databases *GO.db*, KEGG, and *reactome.db*. In addition to enrichment analysis, this tutorial also describes various R functions for visualization of the enrichment results. For easy reading and practicing, all executable R codes/commands start in a separate line with an indent and are highlighted in red text. Those red codes are intended to be typed in RStudio *Console* pane and practiced by the readers. Following R convention, comments on codes/scripts are indicated with a leading pound sign *#* immediately after the corresponding codes. R functions, operators, and arguments mentioned in the text part (not the code part) are highlighted in italicized blue text. R package names and other R terms are italicized only.

This tutorial is designed in a way that bench scientists with no experience in R programming can follow. The audience can learn both FunCEA and R skills by practicing this tutorial. I thus try to use plain language and provide detailed annotations to the R codes, especially when a new R term or concept first appears. Those who have no prior R experience may be more comfortable to learn the introduced skills here after they quickly learn one or both R-related tutorials from the same author [12,13]. I always believe that the most efficient learning is achieved by doing. It is thus advised that the audience type the provided codes and review the code outputs in RStudio, rather than just reading the code texts. I will include some code output as screenshots or figures, but most of the time readers should check the code outputs/results on R screens of different panes (i.e., panes of *Console*, *Plots*, *Environment*, and *Data Viewer*). The key to conducting FunCEA in R is a good understanding and proficient use of R functions and other basic R concepts. For this purpose, this tutorial includes Appendix A to introduce the basics of R functions, data frames, vectors, and objects. Finally, an R Markdown file with codes for the major steps and its rendered HTML version are included as Supplementary Materials of this tutorial. The HTML output file include codes output and plots, which help the audiences better understand FunCEA.

## 2. Materials and Equipment

### 2.1. Equipment

A desktop or laptop computer (either PC or Mac). Internet access is needed since *clusterProfiler* directly interrogates the KEGG knowledgebase via internet. Internet connection is also essential to install R, RStudio, and the relevant R packages.

### 2.2. Sample Data

RNA-seq statistical result in comma separated values (CSV) format is used as the starting data. Starting with this real-world data rather than a pre-prepared gene list from a package benefits beginners. It is available as the Supplementary Materials with a file name of OE_vs_KO.csv.

### 2.3. Software

No commercial software is needed. This tutorial uses R (V4.5), RStudio (2025.5.0.496), and the relevant R external packages (*clusterProfiler*, *org.Hs.eg.db*, *enrichplot*, *AnnotationDbi*, *ggplot2*, *dplyr*, *ReactomePA*, *GO.db*, *ggtangle*, and others). All are open source and free to use. The versions of major packages used in this tutorial were *clusterProfiler* (V4.16), *org.Hs.eg.db* (V3.21), *ReactomePA* (V1.52), *GO.db* (V3.21), *enrichplot* (V1.28.4), and *AnnotationDbi* (V1.70). 

## 3. Raw Data and Its Import to R

Before analysis, install R (https://www.r-project.org/) and RStudio (https://posit.co/download/rstudio-desktop/). Make sure you install R before RStudio. If you encounter any issues installing R or RStudio, please consult your IT support team or any knowledgeable and supportive person.

We use results of RNA sequencing (RNA-seq) as an example for biological FunCEA of a gene list (see Supplementary Data). The file name of the DESeq2 [14] output is *OE_vs_KO.csv*. This RNA-seq project studied the role of human *BRD2* in induced pluripotent stem cell (iPSC) reprogramming in the author’s lab [15]. We compared the transcriptional differences between *BRD2* overexpression (OE) and knockout (KO) in human fibroblasts that undergo iPSC reprogramming. You can create a new folder of *classEnrichment* and save *OE_vs_KO.csv* in the *classEnrichment* folder (directory in R terminology). Set this folder as the working directory (WD) using the base R *setwd()* (stands for “set working directory”) function (see Appendix A.1 for a description of R functions) in the RStudio *Console* pane (the lower-left pane as its default location). Type the following code in the *Console* pane and then hit the *Enter* key (like this code, you should hit the *Enter* key to execute all other codes in red text in this tutorial, which is not explicitly stated in all the codes),

setwd(“full_path2classEnrichment”) # Set the folder where the *OE_vs_KO.csv* file is located as the R working directory. You need to use your full path, which is different from the path description I wrote here.

**Figure 2:**
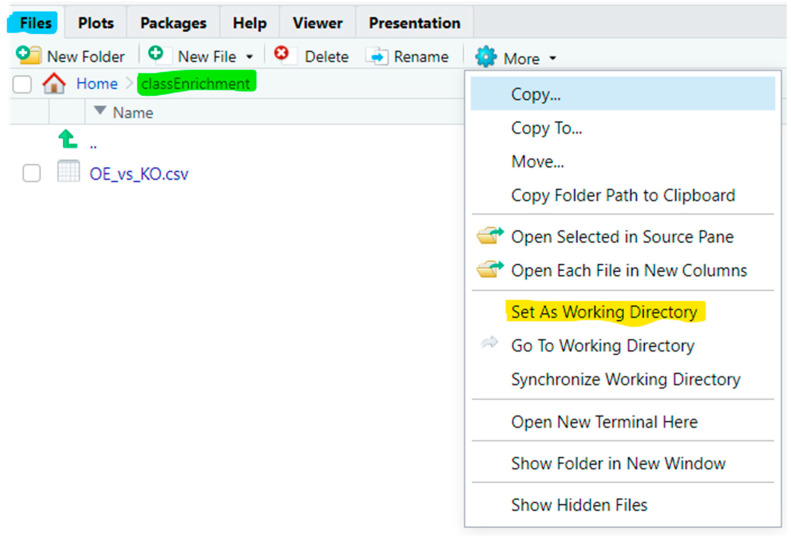
GUI method for setting working directory from the *Files* pane. Target folder is highlighted in green, and action is highlighted in yellow. The corresponding code will show in the *Console* pane. The *Files* pane tab is highlighted in cyan.

Alternatively, in the *Files* pane (the lower-right pane as its default location in RStudio), navigate to the *classEnrichment* folder, and then click on “*More*” cog tab in the tab bar of the *Files* pane to bring about the pulldown menu (Figure 2). Click “*Set As Working Directory*” on the pulldown menu. On the *Console* pane, you can see the corresponding full command line for the same action.

After setting up the working directory, you can see the full path to your working directory by executing,

getwd() # This function means “get working directory”. The full path will be printed out in the *Console* pane.

Upon setting the folder with your file as the working directory, you can list and see the files using the *list.files()* function. Just run,

list.files() # This command prints out the files in the current working directory onto the *Console* screen.

Read in the RNA-seq results data into R using the base R function *read.csv()* and put the data in an R object named *rawData* (see Appendix A.4 for a brief introduction to R objects). To bring about the built-in help page of *read.csv()*, type and run ?read.csv or execute help(read.csv) in the RStudio *Console*. The help page will show in the *Help* pane. For other functions in this tutorial, you can find the help pages using *?* or *help()* function as you do for *read.csv()* here.

rawData <- read.csv(“OE_vs_KO.csv”) # The *rawData* object will show on the *Environment* pane in the upper-right quadrant (refer to Appendix A.4 for R objects). The file name should be quoted. This code reads the file into R from the working directory. You can read in a file from any other directory when the full path to the file is included.

The *rawData* here looks like a common table but is a data frame in R terminology, which is a two-dimensional data structure like an R matrix (see Appendix A.2 for a description of data frames and matrices). You can test whether an R object is a data frame with the R test function *is.data.frame()*,

is.data.frame(rawData) # This command should return *TRUE*, an R logical value.

Review the data in the *Data Viewer* pane (upper-left pane by default) by executing,

View(rawData) # This can also be achieved by clicking the *rawData* object on the *Environment* pane.

dim(rawData) # It reveals the number of rows and columns using the dimension function *dim()*. As shown, the data frame contains 35,953 rows and 14 columns.

In the *Data Viewer* pane (in the upper-left quadrant by default), you can scroll down and up, left and right to review the data. You can also sort the data based on any column by clicking the column name.

Or, you can know the entire table structure by printing out the first 2 rows with the column names,

head(rawData, n = 2)

## 4. Install R Packages for Functional Class Enrichment Analysis

The functions used in Section 3 above are from R base packages. We need several external R *packages* to conduct FunCEA. An R *package* is a standardized collection of R codes, functions, sample data, built-in documentation (e.g., help pages and user manual, i.e., vignette in R terminology), or a compilation of knowledge with associated methods. The R core team develops the R base packages, but most R packages are user-developed external ones, which extend R functionality. There are two groups of external R packages for FunCEA: software or tool packages for analysis or visualization, and knowledgebase packages. Knowledgebases for this tutorial include *org.Hs.eg.db*, *reactome.db*, and *GO.db*. The package *clusterProfiler* can interrogate the KEGG web database directly using its built-in functions like *bitr()*, *bitr_kegg()*, and *search_kegg_organism()*. This way, you use the mostly updated KEGG knowledge database and no KEGG annotation package is needed in R. The software/tool packages include *clusterProfiler*, *enrichplot*, and *ReactomePA*. The *clusterProfiler* package provides many functions for FunCEA while *enrichplot* includes many functions for enrichment visualization. Those packages are deposited in the Bioconductor repository. When you install *ReactomePA*, *reactome.db* is automatically installed. The *GO.db* package is required for GO enrichment analysis, but one does not need to explicitly load it.

We use the *BiocManager::install()* function from the BiocManager package to install packages from the Bioconductor repository. However, the BiocManager package itself is installed using the base R package installation function of *install.packages()*. If BiocManager package is not installed, you need to install this tool first,

install.packages(“BiocManager”) # You will see *BiocManager* is now added in your *Packages* pane.

BiocManager::install(version = “3.22”) # You can see now the *biocVersion* package in the *Packages* pane after this command. The version may not be *3.22* at the time you install it; *3.22* is the version when this tutorial was written. Make sure your BiocManager version matches the R version.

Next, we install the annotation tool package *AnnotationDbi* from the Bioconductor repository,

BiocManager::install(“AnnotationDbi”) # When you install *AnnotationDbi*, a lot of dependencies/imports packages are automatically installed, including the following core dependencies: *BiocGenerics*, *Biobase*, *S4Vectors*, *IRanges*, and *RSQLite*.

The following codes represent formats for installing packages from Bioconductor. The first line tests whether *BiocManager* is installed or not using the *if()* statement. The *require()* function within *if()* acts similarly to *library()*. When *!require(“BiocManager, quietly = TRUE)* returns a *TRUE*, which means BiocManager is not installed, the second line installs the *BiocManager* package. Otherwise, i.e., a *FALSE* is returned by *!require(“BiocManager, quietly = TRUE)*, the second line is skipped. The remaining lines install each *Bioconductor* package using the *BiocManager::install()* function.

if (!require(“BiocManager”, quietly = TRUE)) # This script tests whether *BiocManager* is installed or not. The exclamation mark “*!*” here is a logical “not” operator, which means “negate”. Run *!require(“BiocManager, quietly = TRUE)* to see its return/result.

install.packages(“BiocManager”) # It installs *BiocManager* if it is not installed as tested in the first line.

BiocManager::install(“*clusterProfiler*”) # This installs *clusterProfiler* from Bioconductor repository.

BiocManager::install(“enrichplot”) # This installs *enrichplot* from Bioconductor repository.

BiocManager::install(c(“ReactomePA”, “org.Hs.eg.db”, “GO.db”)) # Multiple packages can be installed with one R script when you put them in a vector as shown here for packages of *ReactomePA*, *org.Hs.eg.db*, and *GO.db*. The base R *c()* function generates an R vector (see Appendix A.3).

## 5. ORA for GO Enrichment Analysis

### 5.1. Preparation of Gene Lists for ORA Analysis

For ORA enrichment analysis, we focus on the genes that are differentially expressed. ORA input gene lists include two types: one is the up-regulated and the other is the down-regulated. We usually analyze the two lists separately. ORA analysis just needs the gene IDs in an R vector (a gene list in plain language) as input. R vectors are one-dimensional structure of the same type of data (see Appendix A.3). The gene list may and may not be ranked, and a bare list of genes without the associated expression values works.

First, subset the up-regulated data using the base R *subset()* function,

sigUpData <- subset(rawData, subset = log2FoldChange > 1 & padj < 0.05)

The above script subsets genes that are significantly up-regulated by at least 2-fold at the significant level of padj < 0.05. It uses two criteria to subset the data with the logical operator *&* as shown in this code. Those rows (genes, or transcripts) that meet both conditions are kept. This code keeps all columns. For details of *subset()* function, find its help page by issuing help(subset) or ?subset in RStudio *Console* pane. For a general introduction of R functions, see Appendix A.1. We store the resulting small data frame (less rows) in an R object *sigUpData* using the assignment operator *<-*. R objects are stored temporarily on your computer active memory that is available for subsequent use during your R session. Otherwise, the resulting small data frame is just printed out on your screen and cannot be used in the following analysis.

You can check the dimension of the resulting data frame,

dim(sigUpData) # The dimension function *dim()* reveals that there are 775 rows only now. The dimensional information is also displayed in the *Environment* pane.

Similarly, we can subset data for the down-regulated genes,

sigDownData <- subset(rawData, subset = log2FoldChange < −1 & padj < 0.05) # This code subsets genes that are significantly down-regulated by at least 2-fold at the significant level of *padj < 0.05*. The logical operator *&* is used in this code to define two criteria for subsetting.

The above code uses two conditions to subset: one is the *log2FoldChange* and the other is the *padj* values. It just uses the column name to define conditions for subsetting for the selected columns. When two criteria (two columns) are used to subset the data frame, you use the logical operator *&* to indicate that the two conditions must be met as shown above. Please note that the thresholds are arbitrary depending on your projects. In this protocol, we use 2-fold as the threshold, i.e., *log2(fold change) > 1*, or *log2(fold change) < −1*.

You can review *sigUpData* in the *Data Viewer* pane (upper-left) by clicking *sigUpData* in the *Environment* pane, or just issue,

View(sigUpData) # View the subsetted smaller data frame in the *Data Viewer* pane.

As you can see, the first column is ENSEMBL IDs. The ENSEMBL IDs in this column will be our input gene list for GO ORA. You can extract this gene list as follows,

sigUpGeneList <- sigUpData$ENSEMBL # It uses the extraction operator *$* along column name to extract one column of data from a data frame as a vector as demonstrated here.

Alternatively, you can generate the gene list using the square bracket extraction operator *[ ]* by either index (position of the column) or column name with a syntax of *[row, column]*.

sigUpGeneList1 <- sigUpData[, 1] # Extract by index. ENSEMBL is column 1. The comma without a row number here indicates that all rows are extracted to the new data frame (multiple columns) or the resulting vector (when one column extracted). Because the *drop* argument defaults to *TRUE*, the resulting data are a vector. It will be one column data frame when its value is defined as *FALSE*, i.e., *sigUpData[, 1, drop = FALSE]*.

sigUpGeneList2 <- sigUpData[, “ENSEMBL”] # Extract by column name, i.e., ENSEMBL.

The above three gene lists are identical, and those can be tested using the R test function *identical()*,

identical(sigUpGeneList, sigUpGeneList1) # The return is *TRUE*.

identical(sigUpGeneList, sigUpGeneList2) # The answer is *TRUE*.

Similarly, you can generate the list of genes down-regulated by *BRD2*,

sigDownGeneList <- sigDownData$ENSEMBL # Or, use sigDownData[, “ENSEMBL”]

### 5.2. Load the Required Packages

With the gene list generated, we can profile the genes for GO term enrichment using the function of *enrichGO()* from the *clusterProfiler* package. First, we need to load the tool package *clusterProfiler* using the *library()* function,

library(*clusterProfiler*) # You can also load any package by clicking the box on the left of the package name in the RStudio *Packages* pane.

To see the original literature of *clusterProfiler*, you use the *citation()* R function,

citation(“*clusterProfiler*”) # This command lists the original publications from Dr. Yu’s group.

To find out the package manual/vignettes,

browseVignettes(“*clusterProfiler*”) # It pops up a window containing the hyperlink for the package documentation in the default browser. Clicking the *HTML* will lead to the landing page that includes hyperlinks of different topics, including the vignette.

To conduct GO enrichment analysis, we also need the knowledgebase package. Only the organism-level annotation package needs to be loaded. *GO.db* should be installed, but may not be loaded. Since human genes are analyzed here, we load the *org.Hs.eg.db* package,

library(org.Hs.eg.db) # This also loads the basic *AnnotationDbi* and other required Bioconductor and annotation packages (*Biobase*, *generics*, *BiocGenerics*, *IRanges*, *S4Vectors*, and *stats4*). Other organisms have their own organism-level annotation packages; for example, you install and load *org.Mm.eg.db* if the genes in your input gene list are mouse ones.

### 5.3. Generate the Enrichment Object Using the enrichGO() Function

Gene ontology (GO) is commonly analyzed in FunCEA. GO has three sub-ontologies: biological process (BP), molecular function (MF), and cellular component (CC). *clusterProfiler* package provides functions to conduct GO enrichment analysis. The *enrichGO()* function conducts the traditional ORA of gene ontology.

The simplified syntax for *enrichGO()* with the first four arguments is as follows.

*enrichGO(gene, OrgDb, keyType = “ENTREZID”, ont = “MF”)* # This is not a code for practice. It is the syntax.

The full syntax can be found on the built-in help page, which can be pulled out by running,

help(enrichGO) # Or, simply execute ?enrichGO.

The above syntax includes four basic arguments (parameters in plain language). The first argument *gene* defines the list of input genes; *OrgDb* defines the organism-level annotation package; *keyType* defines the type of gene ID with *ENTREZID* as the default; and *ont* defines GO category, which could be *BP*, *CC*, *MF*, or *ALL* (*MF* as the default). The first and the second arguments (*gene* and *OrgDb*) have no default values. These arguments are called *required arguments*, for which users need to provide values. For an introduction of R function and its arguments, refer to Appendix A.1.

Let us analyze *BP* enrichment for the human gene list of *sigDownGeneList*,

egoBP_down <- enrichGO(gene = sigDownGeneList, OrgDb = org.Hs.eg.db, keyType = “ENSEMBL”, ont = “BP”) # The results are stored in *egoBP_down* object. Since our gene list uses ENSEMBL IDs, we change the *keyType* setting from the default “*ENTREZID*” to “*ENSEMBL*”. When you want to analyze biological process, you change the *ont* setting from the default “*MF*” to “*BP*”.

The argument names can be omitted if you position all of the values in the order shown in official syntax (function definition order). When the argument names are omitted, the order of the arguments is strictly enforced. When all of the argument names are provided, the order of arguments inside the parentheses of the function does not matter. The above code can be simplified as,

egoBP_down <- enrichGO(sigDownGeneList, org.Hs.eg.db, “ENSEMBL”, “BP”) # This simplified code works, but it would not work when the order of the argument values would be changed. Readers are encouraged to change the positions of the four argument values here and experience what happens.

### 5.4. Review and Extract the Enrichment Results as a Traditional Data Frame

The resulting enrichment object (*egoBP_down*) is an R S4 data structure, which is not immediately human-accessible or readable.

isS4(egoBP_down) # The *isS4()* function tests whether it is an S4 object.

You can have an overview of the results by simply issuing the object name,

egoBP_down # This is implicit use of print(egoBP_down).

**Figure 3:**
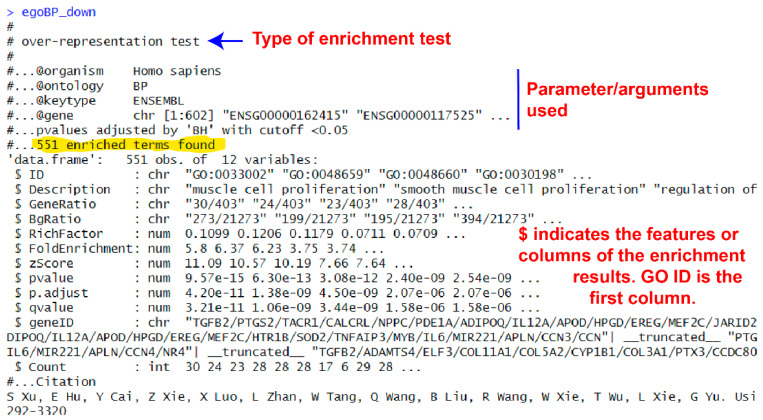
Screenshot for overview of enrichment results. The author adds annotations in red text and yellow highlight.

The above code prints out an overview of the results. You can see information about the analysis; for example, type of analysis, argument values used, and summary of enrichment. In this example, it shows “551 enriched terms found” at the time of writing (the number of enriched terms may vary when the tutorial will be used due to update of knowledge databases). The enrichment statistics include 12 features that are present in 12 columns in the result data frame. When the contents are long for any item, only the first several items are printed out (see Figure 3).

The primary elements of an R S4 object (data structure) are *slots*. You can list the names of slots in an S4 object using the *slotNames()* function,

slotNames(egoBP_down)

You can see that there are 15 slots and the first one is *result*. We can access S4 object *slots* using the @ S4 accessor. Let us extract the enrichment analysis *result* slot,

egoBP_downRes <- egoBP_down@result # This extracts the enrichment statistics results as a data frame using the @ operator from the *result* slot.

View(egoBP_downRes) # The *View()* function allows you to review the results in the *Data Viewer* pane. You can just click *eogBP_downRes* in the *Environment* pane to bring its contents to the *Data Viewer* pane.

Now, the results in the resulting data frame are easy to read and understand. You can scroll up and down the table or left and right in the table to review. You can sort the values of any column by clicking the column name.

The resulting data frame (table in plain language) includes all results (enriched and non-enriched). If you just want to generate a data frame for the enriched terms, use the following code,

egoBP_downEnrichedRes <- as.data.frame(egoBP_down) # The *as.data.frame()* function extracts result for the enriched terms only as a data frame.

The results obtained by the above two methods (@ accessor or *as.data.frame()*) are both traditional data frames, which can be processed by functions for the conventional data frames.

View(egoBP_downEnrichedRes) # Review results in *Data Viewer* for the enriched terms.

You can see the differences in number of terms in the two result data frames above using the dimension function *dim()*,

dim(egoBP_downRes) # As you can see, there are 4386 rows/terms for this data frame.

dim(egoBP_downEnrichedRes) # As you can see, there are 551 rows/terms for this data frame. Each row is an enriched GO term of the biological process aspect.

The above two data frames (table) each include 12 features (columns) for each enriched term. Those column names can be listed using,

names(egoBP_downEnrichedRes) # This is equivalent to colnames(egoBP_downEnrichedRes).

Conveniently, we can treat the *clusterProfiler* S4 result object like a data frame and use the conventional data frame functions/methods to access or manipulate data of the *result* slot like *dim()*, *head()*, *[ ]* extract operator, and others. For example,

We can subset the *result* slot (a data frame) directly from the S4 object (not the extracted data frame),

egoBP_downPadjust005 <- subset(egoBP_down, egoBP_down$p.adjust < 0.05) # This code subsets the enriched terms with the *p.adjust* threshold of 0.05. The resulting data frame is identical to *egoBP_downEnrichedRes* and one can test it with identical(egoBP_downEnrichedRes, egoBP_downPadjust005).

To subset row 1 to 10 of the results directly from the S4 object using the conventional data frame extraction operator *[ ]*,

top10terms <- egoBP_down [1:10] # This extracts the first 10 rows of the enrichment *result* slot (slot 1).

### 5.5. Remove the Redundant Terms

As you can see, there are 551 enriched BP terms for the down-regulated genes by *BRD2* at the time of writing (the number may change when you practice this tutorial due to updated new databases). Many of those terms are redundant. *ClusterProfiler* provides the *simplify()* function to remove the redundant terms.

nrow(egoBP_downRes) # This reveals the total number of terms that are analyzed, which is 4386.

nrow(egoBP_downEnrichedRes) # This code reveals the number of enriched terms.

egoBP_downSim <- *clusterProfiler*::simplify(egoBP_down) # This code uses the default settings to remove the redundant terms. We use *clusterProfiler::simplify()* here to avoid namespace conflict because *igraph* and *purr* also have a *simplify()* function.

egoBP_downSimRes <- egoBP_downSim@result # This extracts the statistical results for the enriched terms.

egoBP_downSimEnrichedRes <- as.data.frame(egoBP_downSim) # It converts the enriched result to a data frame.

nrow(egoBP_downSimRes) # As you can see, the number of terms becomes 241 after removing the redundant terms.

nrow(egoBP_downSimEnrichedRes) # The number of enriched terms is also 241, as revealed by this code.

Please note that *simplify()* returns the enrichment results for the enriched terms only. The help page of *simplify()* reads “simplify output from enrichGO and gseGO by removing redundancy of enriched GO terms”. Therefore, the data frame prepared by slot extraction via the @ S4 accessor and by *as.data.frame()* are the same. You can test this fact by,

identical(egoBP_downSimRes, egoBP_downSimEnrichedRes) # As you can see, the answer is *TRUE*.

### 5.6. Extract the Experimental Gene List for an Enriched Term

You may want to see the full list of experimental gene IDs that fall into a specific enriched term. Or, you may want to generate a list of the experimental genes that fall into a group of closely related terms. How is this achieved? This information is on the *geneID* column (or the 11th column) of the *result* data frame. We have several ways to extract this information.

#### 5.6.1. Extract Experimental Genes of a Term Directly from the S4 Results Object Using Methods for the Traditional Data Frame

First, we use simple indexing methods to extract directly from the enrichment object *egoBP_downSim*.

TermGeneString1 <- egoBP_downSim[4][“geneID”] # This extracts the value of the fourth row/term in the *geneID* column, i.e., gene ID for the fourth enriched term. It results in a one-column/one-row data frame.

The above code extracts gene IDs directly from the S4 object for the enriched term at row 4 in the internal results data frame. To find out in which row a term is located, one can use the *which()* function with the following code,

which(egoBP_downSimRes$Description == “response to ethanol”) # This code returns a number, which is the location (index) of the enriched term of “response to ethanol”. The *Description* column of the internal results data frame includes each of the enriched terms. The *==* is relational operator and it tests whether the two on its left and right sides are the same. It has returns of *TRUE* or *FALSE*.

The above code finds out the row number for the BP term of “response to ethanol”, which is indicated in the *Description* column. Or you can use the following code to get the same result,

which(egoBP_downSim@result$Description == “response to ethanol”) # This code first extracts the data frame from the *result* slot and then tests each value of the *Description* column of the resulting data frame on the string of “response to ethanol”. The returns are *TRUE* where there is term of “response to ethanol”. All other terms in the *Description* column have a *FALSE* value for the test.

#### 5.6.2. We Can Also Extract the Gene ID Information from the Result Data Frame

TermGeneString2 <- egoBP_downSimRes[egoBP_downSimRes$Description == “response to ethanol”,][“geneID”] # The result is a one-column/one-row data frame.

The above code uses the extraction operator *[ ]* two times. The first time, it subsets the row where “response to ethanol” is located. This first step results in a one-row data frame. The comma here means that all columns will be kept/extracted. The next step subsets the *geneID* column from the resulting one-row data frame. The second step can also use the *$* extraction operator like this,

TermGeneString3 <- egoBP_downSimRes[egoBP_downSimRes$Description == “response to ethanol”,]$geneID # However, this results in a vector.

Test if those are the same:

identical(TermGeneString1, TermGeneString2) # The answer is *TRUE* because both are data frames.

identical(TermGeneString1, TermGeneString3) # The answer is *FALSE* because one is a vector and the other is a data frame.

To convert the one-row-one-column data frame to a vector, you extract the values in the *geneID* column with *$*,

TermGeneString <- TermGeneString1$geneID # Now, you generate a vector with this code.

identical(TermGeneString, TermGeneString3) # Now, the answer is *TRUE*. You can directly test it like this: identical(TermGeneString1$geneID, TermGeneString3).

### 5.7. Convert the One-String Gene List to a List of Individual Genes

The result above puts all genes in one R string. In other words, all gene ID for a GO term is in one R string separated by /. How do we make it a vector of the gene list?

We can use *strsplit()* function from the base R to make it a vector (a list of genes),

TermGenesList <- strsplit(TermGeneString3, split = “/”) # It generates a vector of genes in the format of an R list.

Print out the two to compare and see the differences between the two outputs,

TermGenesList

TermGeneString3

You can generate the vector of genes for an enriched GO term in one R script/code using base R pipe operator *|>*,

egoBP_downSimRes[egoBP_downSimRes$Description == “response to ethanol”,]$geneID |> strsplit(split = “/”) -> TermGenesList2

The above code uses the pipe operator *|>*. It allows result/output from the previous step/function to be input for the next step/function. At the end of the above code, we use the variant of the assignment operators, ->, to assign the result to an R object, i.e., *TermGenesList2*.

Compare the two gene lists,

identical(TermGenesList, TermGenesList2) # The answer is TRUE.

However, the above object is an R list with one component only,

class(TermGenesList) # The test answer is *list*, a special R data structure different from the common list we know.

length(TermGenesList) # The answer is 1 because it has one R string only in this R list.

However, we know that there is more than one gene in the list. R list is an R data structure to hold heterogenous data in a named list. It is not the generic list as an ordinary person knows. It is structurally different from the “input gene list” mentioned in this tutorial. The “input gene list”, in fact, is an R vector. To convert an R list to an R vector, you use the *unlist()* R function,

TermGenes_vector <- unlist(TermGenesList) # This converts the R list to an R vector.

is.vector(TermGenes_vector) # The answer is *TRUE*.

length(TermGenes_vector) #Now, the output represents how many experimental genes are associated with this enriched term.

### 5.8. Generate a List of Genes from a Group of Related Terms Based on Index

Sometimes, we want to find out the full list of genes for several closely related terms based on term-gene network analysis (introduced later). For example, we can find out which enriched terms contain the keyword of “lipid”. First,

grep(“lipid”, egoBP_downSimRes$Description, value = TRUE) # This reveals the terms containing “lipid”. The simplified *grep()* R syntax is *grep(pattern, x)*, where *x* is a character vector and *pattern* argument defines the string pattern.

Then, find out the index by using the default value for the *value* argument, which is *value = FALSE*.

grep(“lipid”, egoBP_downSimRes$Description) # This return is 46, 53, 56, and 140. When its default value is used, the argument can be omitted in the function code as shown here for *value = FALSE*.

Now, we can generate the list of genes for all enriched terms that contain the keyword of “lipid”,

lipidGenesList <- strsplit(egoBP_downSimRes$geneID[grep(“lipid”, egoBP_downSimRes$Description)], split= “/”) # Note that you can use a vector of indices, say, *c(46, 53, 56, 140)*. I use code here to get the indices just because you may have different indices at the time of practice due to update of software and knowledgebases.

The *lipidGenesList* object is a list with four components; you can make it a vector by *unlist()* it,

class(lipidGenesList) # The answer is *list*.

length(lipidGenesList) # The length is 4 representing 4 terms, although we have more than 4 genes.

lipidGenesList # This prints out the list. As you can see, there are four components.

lipidGenesVector <- unlist(lipidGenesList)

is.vector(lipidGenesVector) # Test if it is an R vector.

length(lipidGenesVector) # Find out number of genes in this category.

Because the resulting gene list is from several related terms, the terms frequently contain shared genes. We need to remove the redundant genes from the R vector. This can be achieved by the *unique()* function.

lipidGenes_unique <- unique(lipidGenesVector)

length(lipidGenes_unique) # As you can see, the length of the vector is shorter now.

lipidGenes_unique # This prints out the genes.

You can extract genes with similar functions by more than one string pattern using the OR operator |,

grep(“lipid|stero”, egoBP_downSimRes$Description, value = TRUE) # This code returns enriched terms that contain the letter string pattern of “stero” or “lipid” in the term R strings. The “stero” pattern captures “cholesterol” and “steroid” in the GO terms. The logical operator | means “or”. The corresponding indices of those terms can be found when the *value* argument is omitted in the *grep()* function here. The gene list for those GO BP terms can then be generated as demonstrated for the “lipid” pattern above.

### 5.9. Save the Enrichment Results onto Hard Drive

The resulting enrichment data frame is stored in an R object (for a description of R objects, see Appendix A.4). R objects are temporarily stored in the active memory and become lost upon exit of your R session. We need to save it onto the local hard drive. We use *write.csv()* function to achieve this,

write.csv(egoBP_downSimRes, file = “egoBP_down_EnrichedTerm.csv”, row.names = FALSE)

The above code saves the file in the working directory. You can save it in any directory when you provide full path for the *file* argument. Now, you can open the saved file in Excel and review it.

Similarly, you can save the entire original results (including enriched and non-enriched terms) as follows,

write.csv(egoBP_downRes, file = “egoBP4downGenes_results.csv”, row.names = FALSE)

You can save the gene list onto your hard drive and review it with Excel. However, it is better to convert the vector to a data frame. The *write.csv()* function is a variant of *write.table()* base R function. Both save tabular data. For details of those functions, issue help(write.csv), or ?write.csv.

write.csv(as.data.frame(lipidGenesVector), file = “LipidGenesList.csv”, row.names = FALSE)

However, you can directly save an R vector using *write.csv()* function because it implicitly coerces an R vector to a data frame,

write.csv(lipidGenesVector, file = “LipidGenesList2.csv”, row.names = FALSE) # The *row.names = FALSE* avoid adding row names in the form of numbers as one column in the output table. Its default value is logical *TRUE*.

## 6. Visualize the Enriched GO Results from ORA

### 6.1. Dot Plot Visualization

Dot plots are widely used to visualize the term enrichment results. The *enrichplot* package includes a *dotplot()* function for this purpose. Three statistics variables, *gene ratio*, *p.adjust* values, and *number of genes*, are visualized with x-axis scale, colors, and dot sizes, respectively. Although *dotplot()* is from *enrichplot*, you do not need to load *enrichplot* package because *dotplot()* function is imported to *clusterProfiler*. The following code uses the default setting to generate the dot plots of *egoBP_downSim*,

dotplot(egoBP_downSim) # It generates a dot plot in the *Plots* pane.

The above code visualizes the top 10 enriched BP terms by default, with gene ratio as the x-axis. The *p.adjust* values are coded by gradient colors, and the number of genes in each term is represented by the size of dots. The top 10 terms are determined by the *p.adjust* values, i.e., the 10 terms with the 10 smallest *p.adjust* values. But the terms are ordered in the plot along Y axis by their gene ratio.

**Figure 4:**
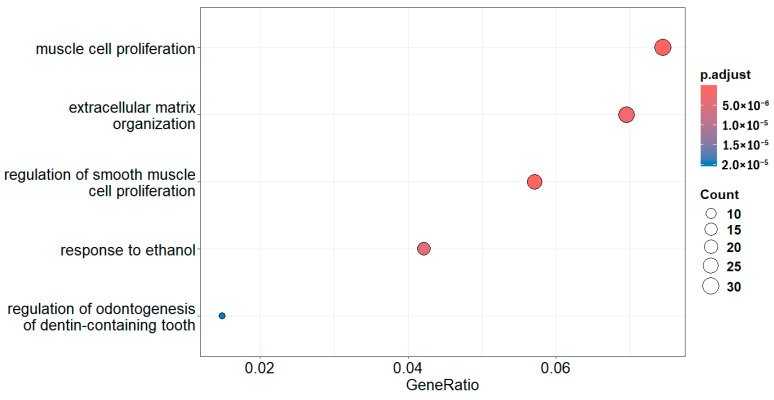
Dot plots for the top 5 enriched BP terms. Number of genes in each term is represented by the size of dots; p.adjust values are expressed by gradient colors. Gene ratio is plotted on x-axis.

You can define how many enriched terms to display by the *showCategory* argument (Figure 4),

dotplot(egoBP_downSim, showCategory = 5) # This code plots the top 5 enriched terms (Figure 4).

You can also define the terms to be visualized by providing a vector of terms to the *showCategory* argument,

dotplot(egoBP_downSim, showCategory = c(“response to ethanol”, “muscle cell proliferation”, “response to nutrient”)) # This code visualizes the three selected terms listed here. You need to use the *showCategory* argument name here because it is not the second argument. Otherwise, you will see an error warning.

Of note, *enrichplot* is built on *ggplot2* and you can use *ggplot2* methods to customize the plots.

**Figure 5:**
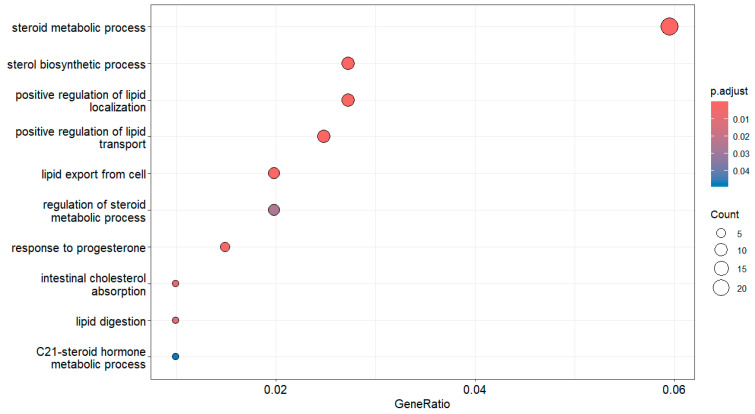
Visualization of lipid-related enriched terms by a character vector of lipid-related terms enriched to the *showCategory* argument.

To generate dot plots for enrichment with keywords of “lipid” and “stero” (e.g., steroid) (Figure 5),

dotplot(egoBP_downSim, showCategory = grep(“lipid|stero”, egoBP_downSim$Description, value = TRUE)) # Since *showCategory* is the fourth argument of *dotplot()*, “showCategory = “cannot be omitted here. For details, find the help page of *dotplot()* by *help(dotplot)*.

### 6.2. Visualize All Three GO Sub-Categories in One Figure as Three Separate Dot Plots

**Figure 6:**
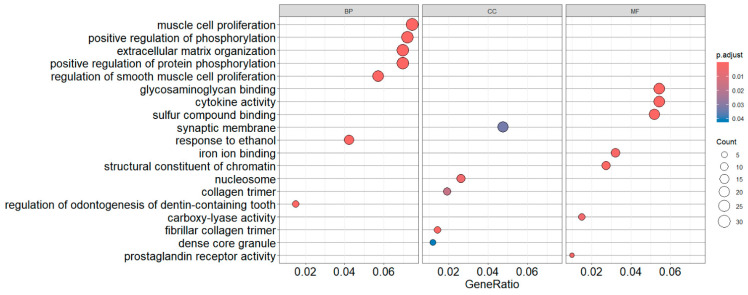
Generate dot plots for the enriched GO terms with separate panels for each sub-ontology.

There are three GO sub-ontologies: *BP*, *CC,* and *MF*. We can conduct enrichment analysis including all three categories and visualize the three in one figure (Figure 6).

First, generate the S4 enrichment object,

egoAllDown <- enrichGO(gene = sigDownGeneList, OrgDb = org.Hs.eg.db, keyType = “ENSEMBL”, ont = “ALL”, readable = TRUE)

egoAllDownSim <- *clusterProfiler*::simplify(egoAllDown) # It removes redundant terms.

library(ggplot2) # The *ggplot2* package is needed for plot customization.

dotplot(egoAllDownSim, showCategory = 7, split = “ONTOLOGY”, label_format = 60) + facet_grid(.~ONTOLOGY) + theme(panel.grid.major.y = element_line(color = “grey50”))

In the code above, you may or may not quote “*.~ONTOLOGY*” inside *facet_grid()*. To make the horizontal lines more visible (Figure 6), we use the *ggplot2 theme()* function to make the lines darker. The different available grey shades can be found in R simply by the *colors()* command. Or, more precisely,

grep(“grey”, colors(), value = TRUE) # This code uses output of *colors()* as input of *grep()* to find colors with a string pattern of “grey”. It generates a list/vector of grey colors available in R.

The above graphics code splits dot plots based on the factors on the column of *ONTOLOGY* in the *result* slot data frame. There are three value/factors in this column, *BP*, *CC,* and *MF*. You can review this column in the Data Viewer pane by issuing,

View(egoAllDownSim@result) # This code directly brings the result data frame to the Data Viewer without generating an R object.

Or, you can directly list the three types of sub-ontologies (BP, CC, and MF) in the *ONTOLOGY* column using the code below,

unique(egoAllDownSim@result$ONTOLOGY) # The *unique()* function lists unique values for the vector from the *ONTOLOGY* column for the result data frame.

### 6.3. Visualize Gene Network of the Enriched Terms

#### 6.3.1. Generating Term-Gene Networks Using Default Settings

**Figure 7:**
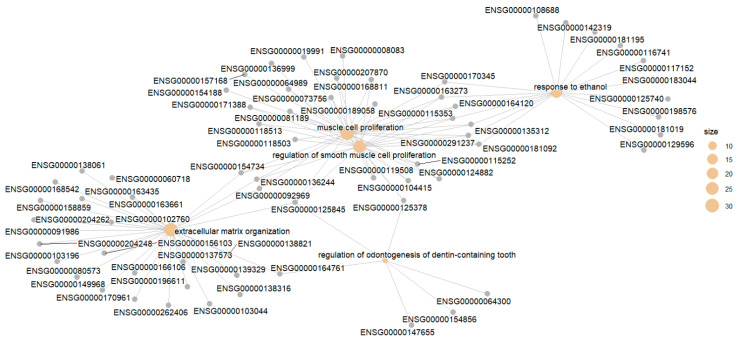
Term-gene network generated by default settings of *cnetplot()*. Each gene is labeled by its ENSEMBL IDs.

The *enrichplot* package has a *cnetplot()* function that can visualize the network of the enriched terms with their associated genes. *cnetplot()* is from *ggtangle* package, but you do not need *ggtangle* since *clusterprofiler* imports it from *ggtangle*. In *ggtangle*, *cnetplot()* means category-item network plot. The following code generates such a network with the default setting (Figure 7),

cnetplot(egoBP_downSim)

The above code visualizes the term-gene network for the top 5 enriched BP terms by default (Figure 7). Each term/category is represented by a colored filled dot (tan, “E5C494” by default), the size of which is proportional to the number of experimental genes in this category. Each unsized grey (by default, “B3B3B3”) dot represents a gene/item in the network.

#### 6.3.2. Label Genes in the Network with Human-Readable Symbol

However, the above code labels each gene with its ENSEMBL ID, which is not human-readable. To solve this problem, we generate enrichment results again and define the *readable* argument as *TRUE*, which has a default value of *FALSE*.

egoBP_downReadable <- enrichGO(gene = sigDownGeneList, OrgDb = org.Hs.eg.db, keyType = “ENSEMBL”, ont = “BP”, readable = TRUE)

egoBP_downReadableSim <- *clusterProfiler* :: simplify(egoBP_downReadable)

**Figure 8:**
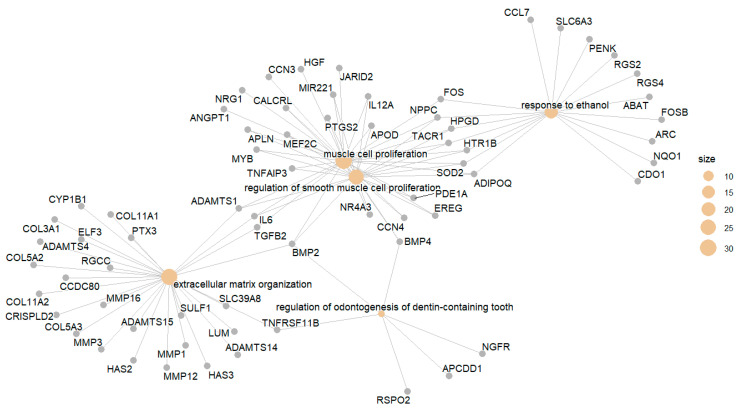
Term-gene network for the suppressed term with human-readable gene labels.

Now, generate the cnetplot again and you can see the genes are labeled with gene symbols (Figure 8), which are human-readable.

cnetplot(egoBP_downReadableSim) # Now, each gene/item is labeled with a human-readable symbol (Figure 8).

#### 6.3.3. You Can Define How Many Top Enriched Terms to Visualize as in *dotplot()*

cnetplot(egoBP_downReadableSim, showCategory = 3) # This code visualizes the term-genes network of the top 3 enriched terms, not top 5 by default.

#### 6.3.4. You Can Also Define Which Terms to Visualize with a Vector of Term Names for the *showCategory* Argument

cnetplot(egoBP_downSim, showCategory = c(“steroid metabolic process”, “sterol biosynthetic process”, “lipid digestion”))

#### 6.3.5. A Quick Way to Visualize the Selected Terms with *grep()* Function

cnetplot(egoBP_downReadableSim, showCategory = grep(“lipid|stero”, egoBP_downReadableSim$Description, value = T)) # This code just visualizes the enriched terms with keywords/string of “*lipid*” or “*stero*” extracted by the *grep()* function. You need to set *value = TRUE* for *grep()*, otherwise the return will be indices not the terms. *showCategory* argument does not work with index.

**Figure 9:**
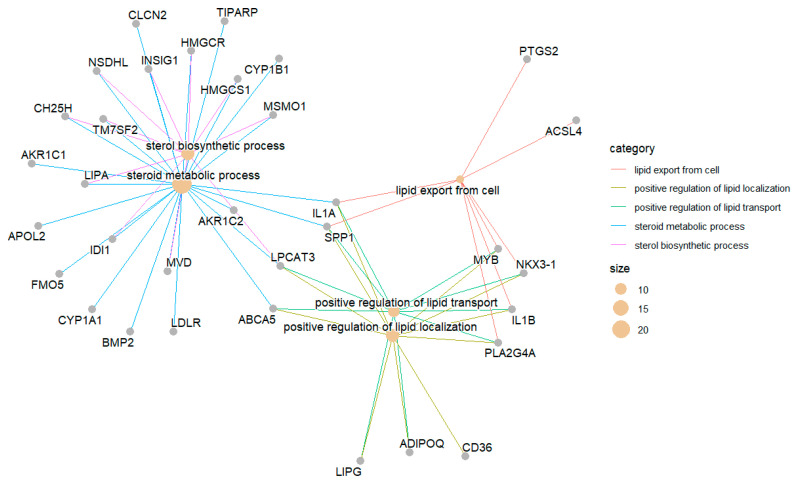
Using colors to distinguish different terms in the term-gene network. The *color_edge* argument allows the edges/lines of different terms to be displayed in different colors.

To enhance visualization, you can use different colors for the edges (the lines linking term to gene) of different terms/category by defining *color_edge = “category”* (Figure 9).

cnetplot(egoBP_downReadableSim, showCategory = grep(“lipid|stero”, egoBP_downReadableSim$Description, value = T)[1:5], color_edge = “category”) # This code just visualizes the first 5 of the enriched lipid terms. This is achieved by subsetting the first 5 terms from the term vector for lipid terms, i.e., *[1:5]*.

You can change the label of the nodes. The default is *node_label = “all”*. Available values are “*none*”, “*category*”, “*item*”, “*exclusive*”, and “*share*”. You can try each preset value and see what happens. The code below uses “*item*”, which is the same as “gene”.

cnetplot(egoBP_downSim, showCategory = c (“steroid metabolic process”, “sterol biosynthetic process”, “lipid digestion”), color_edge = “category”, node_label = “item”) # It works the same if you replace “*item*” with “*gene*” for the *node_label* argument.

#### 6.3.6. Modify the Layout of cnetplot

cnetplot() uses *igraph* package for network layout. You can choose an effective layout to visualize your network by defining the *layout* argument in the *cnetplot()* function. The default is *layout = igraph::layout_nicely*. You can see a pull-down menu when you type *layout = igraph::layout*. You can choose one from the menu and see the effect of the selected layout.

cnetplot(egoBP_downReadableSim, showCategory = 3, color_edge = “category”, node_label = “item”, layout = igraph::layout_with_dh) # As you can see, the layout changes. You can try *layout = igraph::layout.random* and others to see the layout changes.

#### 6.3.7. Visualize Gene Expression Levels in Term-Gene Network Plots

As you can see above, *cnetplot()* visualizes the gene-term network and represents the number of genes in each category with the sizes of the dots, and presents the relationships with edges (lines). In fact, it can also visualize the expression levels by a gradient of colors with the argument of *foldChange*. However, we need the *log2FoldChange* values of each gene for this purpose. To visualize the expression levels, we must generate the gene list in a different way. First, we generate the list with the *log2FoldChange* values. For the purpose of demonstration, we analyze the up-regulated genes (you do similarly for the down-regulated genes).

sigUpGeneListExp <- sigUpData$log2FoldChange # This generates a vector of log2FoldChange for the significantly up-regulated genes.

Then, we name the elements of the vector with the gene IDs using the *names()* function,

names(sigUpGeneListExp) <- sigUpData$ENSEMBL # It names each *log2FoldChange* with its corresponding ENSEMBL ID.

class(sigUpGeneListExp) # As you can see by this test, your data are numeric.

is.vector(sigUpGeneListExp) # This test indicates that you generated an R vector.

sigUpGeneListExp # This prints out the vector, and you can see each element is named with its ENSEMBL ID.

Now, generate the enrichment object for BP GO terms,

egoBP_upExp <- enrichGO(names(sigUpGeneListExp), OrgDb = org.Hs.eg.db, keyType = “ENSEMBL”, ont = “BP”, readable = TRUE) # Please note that you still use the ENSEMBL IDs to conduct the enrichment analysis. Therefore, you extract the element names of the up-regulated gene list with the *names()* function here. Please note that *names()* function can define the names of a vector elements (code above) and can also access/extract element names of a vector (this code).

We remove the redundant terms using the *simplify()* function,

egoBP_upExpSim <- *clusterProfiler* :: simplify(egoBP_upExp)

nrow(egoBP_upExp) # This reveals the number of enriched terms before removing the redundant terms. It can also be achieved by the *dim()* function. Please note that we use the traditional data frame function on S4 object since *clusterProfiler* enables this. 

nrow(egoBP_upExpSim) # This reveals the number of enriched terms after removing the redundant terms. It can be achieved also by *dim()* function.

**Figure 10:**
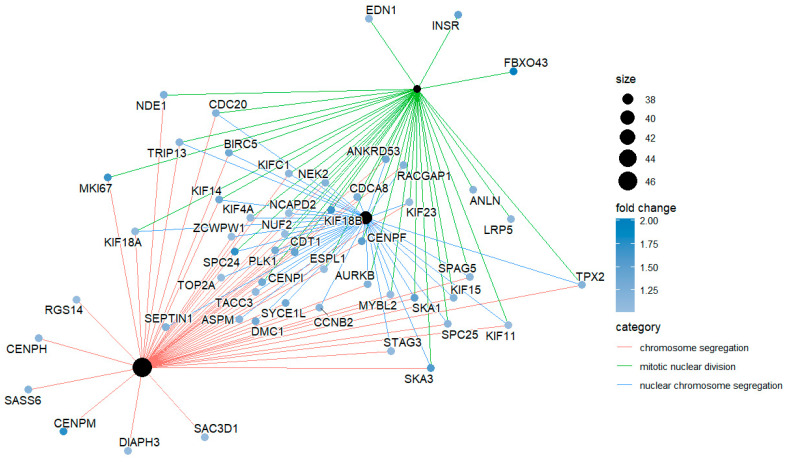
Term-gene network with the expression levels visualized by gradient colors.

Then, generate term-gene network with gene expression levels visualized (Figure 10),

cnetplot(egoBP_upExpSim, showCategory = 3, color_edge = “category”, layout = igraph::layout.davidson.harel, color_category = “black”, node_label = “item”, foldChange = sigUpGeneListExp)

As you can see, this plot additionally includes a color scale for the expression levels, and each gene/item is colored with a shade of colors from the scale (Figure 10). This code sets the color of term/category as “black”, and the edge of each term uses different colors. Since each term can be easily distinguished by its edge color and terms are listed in the legend, we just label each gene and remove the term labels for clarity. Please note that *node_label = “gene”* works the same as *node_label = “item”*. For clarity, we just visualize the top 3 terms (*showCategory = 3*).

Of note, this protocol intentionally uses gene ID list first and uses the expression levels as a list later to show that the list of genes for ORA does not necessarily need the expression levels and the list may not be ranked, which is different from the FCS analysis introduced below.

#### 6.3.8. Using *ggplot2* Function to Customize the cnetplot Network

**Figure 11:**
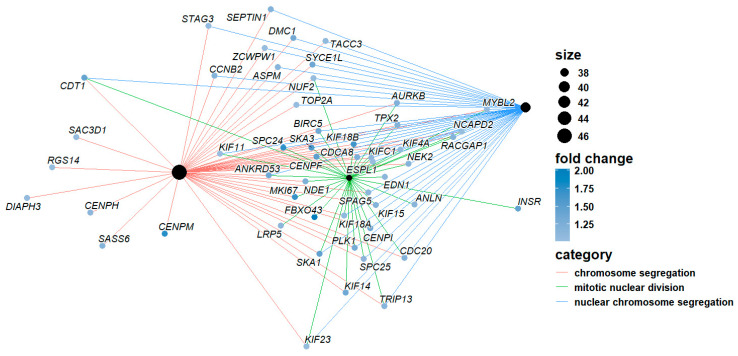
Customization of term-gene network with *ggtangle* and *ggplot2* functions. In this plot, the font sizes and faces for labels, legend title, and legend texts are modified.

The *enrichplot* package is developed on *ggplot2*. Therefore, we can use *ggplot2* methods to enhance the visualization of data such as sizes and face of texts in the plot, and the line thickness.

p <- cnetplot(egoBP_upExpSim, showCategory = 3, color_edge = “category”, layout = igraph::layout.davidson.harel, color_category = “black”, node_label = “none”, foldChange = sigUpGeneListExp, size_edge = 0.7) # This code removes term and gene labels in the plot by *node_label = “none”* so that you can add the node label later with *ggplot2* methods. The default value for *size_edge* is 0.5 and we set it to 0.7 so that the edge line is thicker. We save the plot in an R object *p* so that we can add some features to the plot later using the *ggplot2* methods (Figure 11).

p # As you can see, the plot has no gene and term labels, and the edge line is thicker as compared to Figure 10.

library(ggplot2) # *ggplot2* is needed to add layers to the network plot.

library(ggtangle) # You need to load *ggtangle*. Otherwise, you will see a warning of “*geom_cnet_label() not found*”.

Now, generate the plots with text customization using *ggplot2* layer addition functions,

p + geom_cnet_label(node_label = “item”, size = 4, fontface = “italic”) +

theme(legend.text = element_text(size = 14, face = “bold”),

legend.title = element_text(size = 16, face = “bold”))

*ggplot2* uses plus sign (+) to add layers and components to the plot as shown above. *ggtangle* uses *fontface* argument, but *ggplot2* uses *face* argument for font face. The above code makes the gene symbols italics in the plot via *fontface* argument, and makes the legend and legend title bold via *face* argument of *the element_text()* function.

### 6.4. Generate GO Enrichment Maps Using emapplot() Function

The *enrichplot* package provides the *emapplot()* function that can plot the relationship among the enriched terms. *emapplot()* uses a similarity matrix to generate the enrichment maps. By default, this matrix is not generated when you generate the GO enrichment S4 object.

egoBP_upExpSim@termsim # This code extracts the term similarity slot from the enrichment results S4 object. It returns a 0 × 0 matrix, indicating that the matrix is empty.

We can generate the term similarity matrix using the enrichplot *pairwise_termsim()* function. Unlike *dotplot()* and *cnetplot()*, this function requires explicit loading of the *enrichplot* package.

library(enrichplot)

egoBP_upExpSim <- pairwise_termsim(egoBP_upExpSim) # It generates a term similarity matrix and adds it to the *egoBP_upExpSim* S4 object as a *termsim* slot.

egoBP_upExpSim@termsim # Now, you have a non-empty matrix.

**Figure 12:**
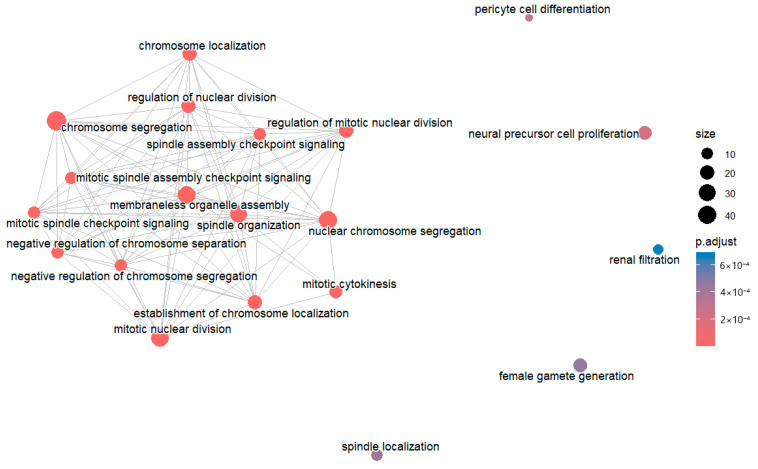
Enrichment map plots to reveal relationships among the enriched terms.

Finally, generate the enrichment map (Figure 12),

emapplot(egoBP_upExpSim, showCategory = 20) # This code plots the enrichment map for the top 20 enriched terms. The default settings plot the top 30 enriched terms. As you can see, some terms are totally independent with no connection with others (Figure 12).

### 6.5. Save Resulting Plots onto Hard Drive

The default R graphics device is the screen. That means that your generated plot appears on the screen in the *Plots* pane. A plot will be lost when a new plot is generated, or when one exits the current R session. How do we save the generated plots onto the hard drive? There are two ways to save a plot onto the hard drive: GUI and file device.

**Figure 13:**
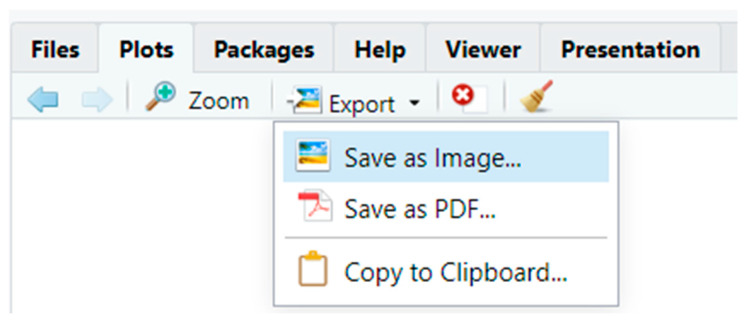
RStudio interface for saving a generated plot to the local drive.

Saving a plot via GUI in RStudio is easy. One just clicks the *Export* tab on the *Plots* pane to bring down the export option menu. From the pull-down menu, you choose “Save as Image” or the other two options to export R plots (Figure 13).

The base R package *grDevices* has several R functions that allow users to save plots to your hard drive in various image formats. Those are *png()*, *jpeg()*, *tiff()*, *bmp()*, and *pdf()*. There are three steps for this purpose. First, open the file device. For example,

tiff(file = “enrichment_map_plot.tiff”) # This opens the graphics file device. When you do not provide the file name here, R will generate a file name of “*Rplot001.tiff*”. Other file devices work the same way. Steps 2 and 3 below are the same for all of the formats.

Second, you generate your plot and the resulting plot will go to the file device, not to the screen device.

emapplot(egoBP_upExpSim, showCategory = 20) # The resulting plot will not show in the *Plots* Pane and is sent to the file device. The file is saved on the current working directory. If you want to save it in any other directory, the full path should be provided in the previous step when you open the file device.

Third, you close the file device to complete the saving,

dev.off() # This step completes the saving of a plot to your hard drive. If this step is missing, the plot file on your hard drive cannot open properly using an external image program.

## 7. ORA Pathway Enrichment Analysis and Visualization

### 7.1. Add Matching Entrez IDs to the Data Frame Before Pathway Analysis

ReactomePA has a function of *enrichPathway()* to analyze pathway enrichment. If you pull out its help page, you will know the input genes should be Entrez IDs since it states that the first argument of “gene” should be “*a vector of entrez id*”. We have ENSEMBL ID in our input RNA-seq data. Because of this, we need to add Entrez IDs to the original data frame. For human gene ID mapping, we need the organism-level annotation package of *org.Hs.eg.db*. Load this package,

library(org.Hs.eg.db) # When mouse genes are analyzed, you should use the *org.Mm.eg.db* package.

Both *select()* and *mapIds()* used below are from *AnnotationDbi* package, but we do not need to load *AnnotationDbi* package explicitly since it is automatically loaded when we load the *org.Hs.eg.db* package.

#### 7.1.1. The select() Method

You can use *AnnotationDbi::select()* function to achieve the same as *mapIds()*, but the procedures are different,

anno <- AnnotationDbi::select(org.Hs.eg.db, keys = rawData$ENSEMBL, keytype = “ENSEMBL”, columns = “ENTREZID”)

However, *select()* function results in 1:many mapping of IDs. The *AnnotationDbi::select()* function generate more ENTREZID and we need to remove the duplicated one later, but *mapIds()* generates the same number of ENTREZ IDs as the original ENSEMBL IDs since the default value for the *multiVals* argument is “*first*”. This can be found by their numbers of rows:

dim(anno)

dim(rawData)

Because of 1:many mapping, we have duplicated ENSEMBL IDs in the *anno* data frame. We remove the duplicated ENSEMBL IDs;

library(dplyr) # The *distinct(), filter(), duplicated()*, and *left_join()* functions used below all need *dplyr* package.

anno <- distinct(anno, ENSEMBL, .keep_all = TRUE) # This can also be achieved by filter(anno, !duplicated(ENSEMBL)).

Now, check the number of rows using either *nrow()* of *dim()*,

nrow(anno)

We need to merge two data frames into one if *select()* is used.

rawDataEntrez <- left_join(rawData, anno, by = “ENSEMBL”) # Since *rawData* and anno have the same number of rows, *left_join()* and *right_join()* work the same.

The resulting data frame with ENTREZID has many NAs in the column of ENTREZID, we need to remove those NAs before enrichment analysis,

rawDataEntrez <- rawDataEntrez[!is.na(rawDataEntrez$ENTREZID),]

Subset the data for DEG based on log2FoldChange and padj, for pathway analysis,

sigUpDataEnt <- subset(rawDataEntrez, log2FoldChange > 1 & padj < 0.05)

sigDownDataEnt <- subset(rawDataEntrez, log2FoldChange < −1 & padj < 0.05)

#### 7.1.2. The mapIds() Method

We first generate a new object for the original rawData,

rawDataEntrez2 <- rawData

We then generate an entrez id vector matching the ENSEMBL IDs,

entrezid <- mapIds(x = org.Hs.eg.db, keys = rawDataEntrez2$ENSEMBL, keytype = “ENSEMBL”, column = “ENTREZID”)

Then, we add this entrez ID vector as a matching column to the original data frame,

rawDataEntrez2$ENTREZID <- entrezid

We remove the *NA* rows,

rawDataEntrez2 <- rawDataEntrez2[!is.na(rawDataEntrez2$ENTREZID) ,]

Although *AnnotationDbi::select()* and *mapIds()* are both data extraction methods of the *AnnotationDbi* package the *select()* function results in a data frame and *mapIds()* outputs a vector. This subtle difference is reflected by their arguments for column selection. *AnnotationDbi::select()* uses *columns*, but *mapIds()* uses *column* because the latter extract one column only. These can be tested as follows,

is.vector(entrezid)

is.vector(anno)

is.data.frame(entrezid)

is.data.frame(anno)

*clusterProfiler* provides the *bitr()* function to convert gene IDs among different ID systems. However, *mapIds()* or *select()* from the *AnnotationDbi* package are generally better and more flexible methods to convert the gene IDs than *bitr()*.

### 7.2. Generate the Gene List

SigDownGeneListEnt <- sigDownDataEnt$log2FoldChange # We can prepare the up-regulated gene list similarly. We use the log2FoldChange values as the list because we can visualize the expression levels later, but it is not necessary. We can directly use the ENTREZID as the list if you will not visualize the expression levels.

names(SigDownGeneListEnt) <- sigDownDataEnt$ENTREZID # This code names each vector element, i.e., log2FoldChange, with its ENTREZID. The gene list may not be ranked.

You can generate the gene list for the up-regulated genes similarly, as shown above, and analyze pathway enrichment the same way as introduced below for the down-regulated genes.

### 7.3. Conduct Pathway Enrichment Analysis

With the gene list prepared, we are ready to analyze pathway enrichment. We need package *ReactomePA*. Load it first,

library(ReactomePA)

Then, analyze pathway enrichment using the function of *enrichmentPathway()*,

ePathDown <- enrichPathway(names(SigDownGeneListEnt), readable = TRUE) # This code uses the default setting for arguments except for *readable*, which is *FALSE* by default. We set *readable = T* so that the gene symbols rather than entrez IDs show up in the category-item network plot using the *cnetplot()* function. We can analyze the up-regulated gene list similarly.

### 7.4. Review the Enrichment Results

To see an overview of the enrichment results, just print the *ePathDown* object,

ePathDown # This is implicit use of *print(ePathDown)*. This allows one to see basic information of the results, including parameters used and number of enriched terms. The output looks similar to what is shown in Figure 3 (See more output images in the supplementary HTML file rendered from the R Markdown file).

isS4(ePathDown) # This tells you that the object *ePathDown* is an S4 object.

slotNames(ePathDown) # List the slot names of the *ePathDown* S4 object.

Generate the full result data frame from the *result* slot using the @ operator,

ePathDownResults <- ePathDown@result # The resulting data frame contains all of the statistical results, including enriched and non-enriched.

Generate the results from *ePathDown* with enriched pathways only,

ePathDownEnriched <- as.data.frame(ePathDown) # The resulting data frame includes the enriched pathways only. This code results in the same results as the code below,

ePathDownEnriched2 <- subset(ePathDown, ePathDown$p.adjust < 0.05)

The above two objects are identical as can be tested below,

identical(ePathDownEnriched, ePathDownEnriched2) # This answer is *TRUE*.

You can review the result on Data Viewer pane by clicking the *ePathDownEnriched* object in the Environment pane, or issue,

View(ePathDownEnriched)

In the *Data Viewer* pane, you can scroll up or down, left or right to review the data. You can also sort the data for a specific column, say, the *qvalue* column, by just clicking the column name.

Save the results as a CSV table onto your hard drive,

write.csv(ePathDownEnriched, file = “EnrichedPathwayDownGenes.csv”) # The full results above can be saved the same way. This saves the file in the working directory. You can save it in any directory when you provide the full path for the *file* argument.

### 7.5. Visualization of the Enriched Results

#### 7.5.1. Dot Plot

The following simple code plots the top 10 enriched pathways.

dotplot(ePathDown)

#### 7.5.2. Category-Item Network Plot

We can use cnetplot() to plot the term-gene (in general term, category-item) network.

cnetplot(ePathDown, foldChange= SigDownGeneListEnt, color_category = “black”, layout = igraph::layout_with_dh, color_edge = “category”, node_label = “gene”, size_edge = 0.8)

For *cnetplot()*, default does not include expression levels (*foldChange* argument as *NULL*). The code here includes fold change values in the plot by a gradient color. The default edge (the line) uses one color (grey by default). Here, each category (term) uses a different color when we set *color_edge = “category”*. The default edge size is 0.5. Here, size_edge = 0.8 makes the lines (both edge and category key lines) thicker. The *node_label* has values of ‘*all*’, ‘*none*’, ‘*category*’, ‘*item*’, ‘*exclusive*’, or ‘*share*’. Here, “gene” is used as *enrichplot* allows for, which is equivalent to “item” in *ggtangle*.

Of course, you can visualize selected terms,

grep(“lipid|stero”, ePathDown$Description, value = TRUE) # It lists pathway terms containing “lipid” or “stero”.

cnetplot(ePathDown, showCategory = grep(“lipid|stero”, ePathDown$Description, value = TRUE), color_edge = “category”, node_label = “gene”, foldChange = SigDownGeneListEnt) # This code generates the network for terms containing “*lipid*” or “*stero*”.

#### 7.5.3. Generate Pathway Enrichment Maps Using emapplot() Function

You cannot generate the enrichment map using the object *ePathDown*; try,

emapplot(ePathDown) # You see the error message because there is no similarity matrix in the term enrichment object.

To generate the enrichment map, we need the term similarity matrix, i.e., *termsim* matrix in the *ePathDown* result object. By default, this matrix is not generated. You can see this using,

ePathDown@termsim # You have a return of <0 × 0 matrix> indicating that it is empty.

library(enrichplot) # Load *enrichplot* to use the *pairwise_termsim()* function below.

ePathDownSimi <- pairwise_termsim(ePathDown) # This will generate the *termsim* matrix and add it to the *ePathDownSimi* object as a slot.

termsimMatrix <- ePathDownSimi@termsim # This extracts the matrix from *ePathDownSimi*.

View(termsimMatrix) # Bring the matrix to the *Data Viewer* pane to review.

Now, you can generate the enrichment map,

emapplot(ePathDownSimi, showCategory = 25) # This code displays map (relationships) for the top 25 enriched pathway terms, and the default is *showCategory = 30*.

## 8. FCS Enrichment Analysis for GO and KEGG Pathways and Visualization

Functional class scoring (FCS) is said to be the second generation of gene function enrichment analysis. It uses different algorithms from that of ORA. FCS aggregates scores of genes in a gene set of each individual controlled term/class along a ranked list of experimental genes. The input gene list needs to be prepared in a different way than the list for ORA introduced above. First, the full gene list should be included. You should not apply any expression threshold to filter out any gene. Second, the gene list should be ranked based on differential expression levels from high to low. In contrast, the input gene list for ORA may or may not be ranked (sorted). Unlike FCS input gene list, as introduced above, the gene list for ORA may or may not include expression levels.

### 8.1. Prepare Ranked Gene List for FCS

#### 8.1.1. Using Base R Function to Generate the Input Gene List

This subsection uses base R functions, and you do not need to load any external R packages.

GeneList4FCS <- rawData$log2FoldChange # Generate the gene list with their corresponding log2(fold changes).

The above code generates a vector of gene list for FCS. Please note that the components are the corresponding log2(Fold Change), not any type of gene IDs. You include all of the data and do not apply an expression level threshold to filter out any gene. Therefore, we do not subset the expression data before generating the gene list.

Then, you name the *GeneList4FCS* with the gene IDs using the generic accessor function *names()*,

names(GeneList4FCS) <- rawData$ENSEMBL # This gives each expression level a name with its corresponding ENSEMBL ID.

Rank the gene list from high to low,

GeneList4FCS <- sort(GeneList4FCS, decreasing = TRUE) # The list should be ordered from high to low.

#### 8.1.2. Tidyverse Way to Generate the Ranked Gene List

We can use one code to generate the input gene list with the *dplyr* package, one of the *tidyverse* packages.

library(dplyr) # You need *dplyr* package since the *arrange()*, *desc()*, and *pull()* functions and the *%>%* pipe operator are from *dplyr*.

GeneList4FCS2 <- rawData %>% arrange(desc(log2FoldChange)) %>% pull(log2FoldChange, name = ENSEMBL) # The %>% pipe operator allows output from the previous step as input of the next step. The *name* argument of the *pull()* function allows elements of the pulled-out vector to be named with the corresponding elements of the provided column. For details, find their help pages using commands of help(arrange), help(desc), and help(pull) in RStudio *Console* pane.

identical(GeneList4FCS, GeneList4FCS2) # The *identical()* function tests whether the two named vectors generated are the same or not. The answer is *TRUE*.

Of note, it is suggested that the DESeq2 shrunken *log2(fold change)* is the preferred expression level for ranking the gene list. If you want to use the shrunken log2(fold change), the results data should be generated using the *lfcShrink()* function in the statistical step using DESeq2.

### 8.2. FCS Enrichment Analysis of Gene Ontology Terms Using the gseGO() Function

library(*clusterProfiler*) # Load the *clusterProfiler* package.

library(org.Hs.eg.db) # The *org.Hs.eg.db* package should be loaded. For mouse genes, use the organism-level annotation package of *org.Mm.eg.db*.

gseBP <- gseGO(geneList = GeneList4FCS, OrgDb = org.Hs.eg.db, keyType = “ENSEMBL”, ont = “BP”)

The above code generates GO enrichment results for *BP* sub-ontology using the FCS algorism. You can conduct FCS enrichment analysis for *CC*, *MF*, or *ALL* by modifying the *ont* argument. The default *keyType* is ENTREZID. We use ENSEMBL here. Unlike ORA using gene ID as the input gene list for enrichment analysis, gene list elements for *gseGO()* are *log2FoldChange*. Possible key types for human annotation can be found by,

keytypes(org.Hs.eg.db) # For mouse gene list analysis, use the annotation package of *org.Mm.eg.db*.

### 8.3. Overview of the Results

You can have an overview of the results by issuing,

gseBP # This is equivalent to print(gseBP).

The above code prints out an overview of the enrichment results, including parameters used for the enrichment analysis, how many terms are enriched, and the 11 variables in the result data frame. Although the literature claims that FCS is more sensitive, this protocol shows that FCS reveals many fewer enriched BP terms (21 vs. at least 241), at least in the case where there is a good list of DEGs. The number of enriched terms varies from run to run even on the same day due to random sampling of genes to calculate the exact p-values by the underlying *fgseaMultilevel()* function (https://github.com/YuLab-SMU/clusterProfiler/issues/763, accessed on 17 January 2026). The resulting object is an S4 object. You can test this by,

isS4(gseBP) # You have a *TRUE* answer.

R S4 object includes slots as its components. To list the slot names of *gseBP*,

slotnNames(gseBP) # The *slotNames()* function lists all the slot names in the S4 *gseBP*.

You can print out or access to each slot contents using the @ operator. For example,

gseBP@params # This prints out the parameters used for enrichment analysis.

gseBP@organism # This tells you what organism the data are about.

gseBP@setType # This reveals what GO type is analyzed. It is *BP* in this example.

gseBP@keytype # It reveals what key type was used.

The main statistical data of enrichment analysis is in the *result* slot. You can see the overview of the *result* slot using the data structure function of *str()*,

str(gseBP@result) # This code gives you an overview of the *result* slot. As you can see from the output, *result* slot is a data frame with 11 variables (columns). The column names (variables) are listed in the output of *str()* as indicated by a *$*. You will have more messy output if you directly use *gseBP@result* without *str()*.

To extract the result slot as a data frame,

gseBP_result <- gseBP@result # This extracts the “*result*” data frame slot for the enriched terms.

You can save the result data frame onto local hard drive and review it with Excel,

write.csv(gseBP_result, file = “GSEA_results4BP.csv”) # This saves the data frame in CSV format into the working directory. It can be saved on any other directory when the full path is provided for the *file* argument.

### 8.4. Various Visualizations of the GSEA Results

#### 8.4.1. Generate Dot Plots of Enriched Terms for Both Up-Regulated and Down-Regulated Genes

library(ggplot2) # *facet_grid()* needs *ggplot2*.

dotplot(gseBP, label_format = 55, font.size = 14, title = “Dot plot for BP GSEA”, split = “.sign”) + facet_grid(.~.sign)

**Figure 14:**
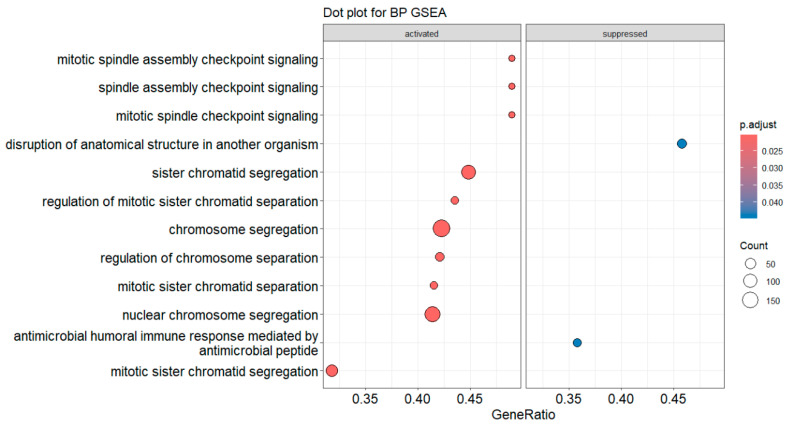
Dot plots with separate panels for the activated and suppressed functions/terms.

The above codes generate dot plots for the top 10 enriched terms by default for both down- and up-regulated genes (Figure 14). The activated and suppressed functions are presented in separate panels. The number of terms can be defined by *showCategory* argument. It generates two dot plots, one for the activated terms and the other for the suppressed terms. The *label_format* argument defines how many characters are in one line of a term label in the dot plot. The default is 30. This code uses 55 so that each term occupies one line only. The *font.size* argument defines the font size for the term names, annotation, and label of the x-axis. Its default size is 12. Please note that there is not a physical column of “*.sign*”. The *dotplot()* function from *enrichplot* package calculates and uses the “*.sign*” values (*+* or *−*) for each term as a non-standard column in generating the dot plots.

#### 8.4.2. Generate Enrichment Maps Using the emapplot() Function

You cannot generate the enrichment map using the original S4 object *gseBP*; try,

emapplot(gseBP) # You see an error message because there is no matrix of term similarity in the S4 enrichment object.

To generate the enrichment map, we need the term similarity matrix, i.e., *termsim* matrix slot in the *gse* results object. By default, this matrix is not generated. You can see this fact using,

gseBP@termsim # It gives a return of *<0 × 0 matrix>*, indicating that the *termsim* slot is empty.

library(enrichplot) # You need to load *enrichplot* to use the *pairwise_termsim()* function below, although one does not need to load it to use the *gseGO()* function.

gseBP2 <- pairwise_termsim(gseBP) # This generates the *termsim* matrix and adds it to the *gseBP2* object.

termsimMatrix <- gseBP2@termsim # This extracts the matrix from *gseBP2*.

View(termsimMatrix) # It brings the matrix to the *Data Viewer* pane to review.

Now, you can generate the enrichment map using the *emapplot()* function,

emapplot(gseBP2) # This code displays term networks for all of the 21 enriched terms (number of enriched terms may vary slightly), including activated and suppressed terms, since the default *showCategory = 30*.

#### 8.4.3. Generate Category-Item Network Plots (cnetplot) with FCS Enrichment Object

##### Generate Network Plot with the SYMBOL Column in the Raw Data

cnetplot(gseBP2) # Use default settings to generate terms-genes network. You can use the *gseBP* object as well.

The above code generates a network with gene IDs of ENSEMBL IDs. Unlike *dotplot()*, the *gseGO()* function does not have a *readable* argument. But we can generate *gse* results with gene SYMBOL as key type.

geneList4FCS_symNA <- rawData$log2FoldChange # Generate a vector of gene expression with log2(Fold Change).

names(geneList4FCS_symNA) <- rawData$SYMBOL # This names vector elements with the corresponding gene SYMBOL.

Note that many rows of the SYMBOL columns have a value of *NA*. We need to remove the *NA* rows using the code below,

geneList4FCS_sym <- geneList4FCS_symNA[!is.na(names(geneList4FCS_symNA))] # This code removes any vector elements whose names are NA using the logical “not” operator of *!* and the test function *is.na()*.

However, there are repeats for some of the SYMBOLs. This is because SYMBOL column is added based on ENSEMBL IDs, and one ENSEMBL ID may result in multiple matches with the same SYMBOL. The following code removes redundant SYMBOLs,

geneList4FCS_symU <- geneList4FCS_sym[unique(names(geneList4FCS_sym))] # This removes redundant SYMBOLs using the *unique()* function and subsetting with *[ ]* operator.

Now, rank the gene list,

geneList4FCS_symU <- sort(geneList4FCS_symU, decreasing = TRUE)

Then, conduct the enrichment analysis,

gseBPsym <- gseGO(geneList = geneList4FCS_symU, OrgDb = org.Hs.eg.db, ont = “BP”, keyType = “SYMBOL”)

Finally, generate the term-gene network with gene symbols as the labels,

*cnetplot(gseBPsym)* # This network labels genes with gene symbols.

Like term-gene networks introduced in previous sections, you can define how many and which terms to display in the plot using the *showCategory* argument.

##### Alternatively, We Can Generate Network Plot via setReadable() Conversion of Gene IDs

The simple way to plot network plots with readable symbols is to use the *setReadable()* function from the DOSE package to generate the enrichment result S4 object that contains gene symbols.

gseBP_readable <- setReadable(gseBP, OrgDb = org.Hs.eg.db, keyType = “ENSEMBL”) # You do not need to load the DOSE package since *clusterProfiler* imports the *setReadable()* function from DOSE.

You can see that the two S4 objects use different gene IDs by extracting the gene IDs for the first enriched term,

gseBP@result$core_enrichment[1] # The gene IDs are in ENSEMBL format with a prefix of “ENSG”. You can simply use gseBP$core_enrichment[1] since *clusterprofiler* treats the enrichment result S4 object like a data frame.

gseBP_readable@result$core_enrichment[1] # The gene IDs are gene symbols, which are human-readable. You can simply use gseBP_readable$core_enrichment[1] to retrieve the same data.

cnetplot(gseBP_readable) # Now the resulting plot has gene symbols as labels of each gene.

#### 8.4.4. Generate Ridge Plots

The *enrichplot* package provides a function of *ridgeplot() *(for details, run command ?ridgeplot). It visualizes enriched terms (gene sets) based on gene density at each expression level for each enriched term. The x-axis is the expression levels (e.g., log2(fold changes)). When the density peak is located on the right side of the x-axis, the gene set/function is activated, whereas if its density peak is located on the left side the term/function is suppressed. In ridgeplot, the *p.adjust* values are visualized by colors in the density plots, i.e., *fill = “p.adjust”* as the default value.

You can just simply generate ridgeplot with all default settings,

ridgeplot(gseBP)

**Figure 15:**
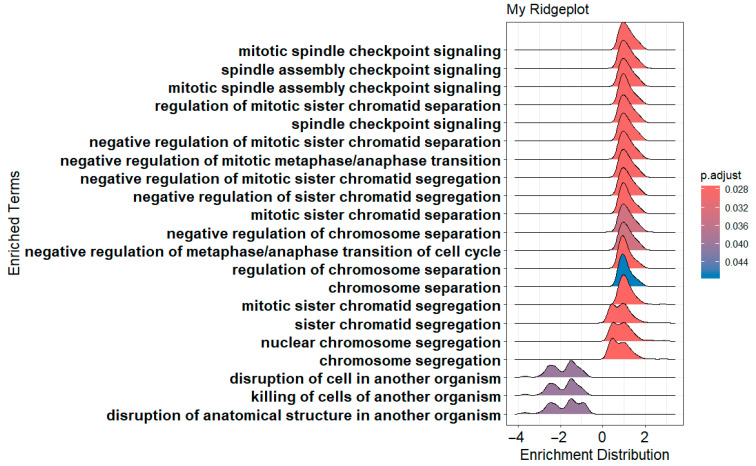
Ridge plot for GSEA enrichment.

You can customize the plot using *ggplot2* methods (Figure 15),

library(ggplot2)

ridgeplot(gseBP, label_format = 70) + labs(x = “Enrichment distribution”, y = “Enriched terms”) + ggtitle(“My ridgeplot”) + theme(axis.text.y = element_text(size = 14, face = “bold”))

In the above code, the *label_format* argument is given a larger number (70 instead of the default of 30) so that each term occupies one line in the plot. The term text size and font are modified with *ggplot2* verbs (Figure 15).

#### 8.4.5. Generate GSEA Plot

**Figure 16:**
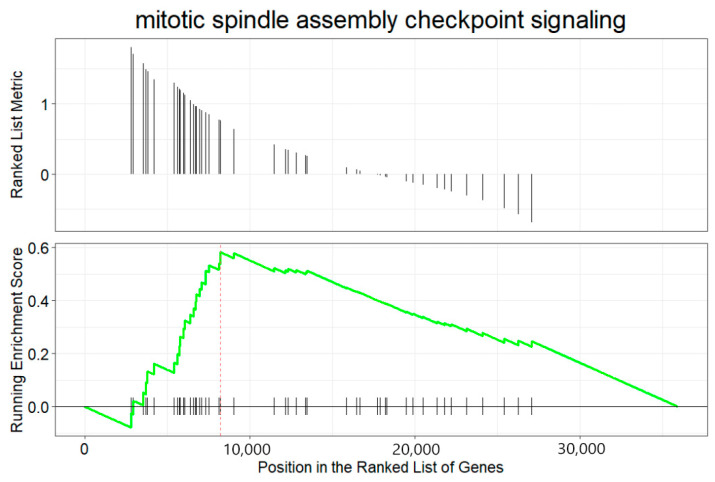
GSEA plot for the enriched term of “mitotic spindle assembly checkpoint signaling”. This function is activated based on the location of the leading edge (the left side of the red line). The green line is the accumulated running enrichment scores.

The *enrichplot* package provides *gseaplot()* function. It plots the running enrichment scores for a specific gene set (term) along the ranked background gene list. GSEA plot usually includes two panels; the upper panel, called Ranked list metric, plots the log2(fold changes) of the members in the selected gene set along the ranked background gene list (Figure 16). The lower panel, called running enrichment score, plots the accumulated running scores along the ranked background gene list. The running enrichment score is a green line, while each member (gene) of the selected gene set (term) is represented as a vertical black bar in both panels. There is a red vertical line in the Running Enrichment Score panel, which represents the maximum running enrichment score (Figure 16). The region beyond this red line is called the leading edge. For the up-regulated (or activated) term, the leading edge is at the left-most side of the red line in the Running Enrichment Score panel, while the leading edge for the down-regulated (suppressed) term is on the right side of the red line in the Running Enrichment Score panel. The background gene list includes all of the genes in your original gene list for GSEA analysis.

gseaplot(gseBP, title = gseBP$Description[1], geneSetID = 1)

The above code visualizes the number 1 enriched term. If you want to visualize the second-most enriched term, just replace the two “1”s in the code with a 2. For the plot title argument, you can define by yourself when you know the term, which should match the *geneSetID*; for example, *title = “mitotic spindle assembly checkpoint signaling”* instead of title = gseBP$Description[1].

## 9. FCS for KEGG Pathway Enrichment Analysis and Visualization

### 9.1. Prepare Gene List When No Allowed Key Type Is Included in the Original Expression Data

*clusterProfiler* provides the *gseKEGG()* function for KEGG pathway enrichment analysis. *gseKEGG()* uses one of the following gene ID systems in the gene list, KEGG ID, NCBI gene ID, NCBI protein IDs, and uniProt protein ID, which are defined by the *keyType* argument of *gseKEGG()* function with values of “*kegg*”, “*ncbi*-*geneid*”, ncbi-*proteinid*”, or “*uniprot*”, respectively.

DESeq2 statistic results include ENSEMBL IDs in its expression analysis, but *gseKEGG()* does not use ENSEMBL ID. See Section 7.1 about how to add ENTREZID to the data frame.

We can now generate the gene list,

geneList4KEGG <- rawDataEntrez$log2FoldChange # This extracts the *log2FoldChange* column as a vector of gene expression.

Name the gene list elements with the corresponding ENTREZID,

names(geneList4KEGG) <- rawDataEntrez$ENTREZID # This gives each *log2FoldChange* value a name with its corresponding ENTREZID. Now, you have a named vector. In plain language, we have a list of *log2FoldChange* values, and each value is named (associated) with its corresponding ENTREZID.

Rank the list from high to low,

geneList4KEGG <- sort(geneList4KEGG, decreasing = T) # This code arranges the log2FoldChange value from high to low using the *sort()* function with a *TRUE* value for its *decreasing* argument.

Each ENTREZID in the named vector of *geneList4KEGG* should be unique. We subset *geneList4KEGG* by its unique names, i.e., ENTREZID with no duplicate,

geneList4KEGGu <- geneList4KEGG[unique(names(geneList4KEGG))] # This removes the duplicated ENTREZID. The “u” here stands for unique.

### 9.2. Generate and Review the Enrichment Results

ePathKEGG <- gseKEGG(geneList4KEGGu, keyType = “ncbi-geneid”)

This code uses default settings except for *keyType*. The default organism is “*hsa*”. For other organism codes, visit https://www.genome.jp/kegg/tables/br08606.html (accessed on 17 January 2026). Upon running the above code, you can see progress message of “Reading KEGG annotation online”. This is because *clusterProfiler* can directly access the KEGG database online. This has two advantages: (1) you always use the most updated knowledge database; (2) you do not need the commercial software for KEGG pathway enrichment analysis, such as the Qiagen Ingenuity Pathway Analysis (IPA).

To see the overview of the *ePathKEGG* results,

print(ePathKEGG) # Or, you just run *ePathKEGG*.

The above code allows you to see the parameters used and the overview of the results, including how many pathways are enriched.

*ePathKEGG* is an S4 object; you can list and see the slot names by,

slotNames(ePathKEGG)

The results are in the first slot, i.e., the *result* slot. You can extract this slot as a data frame with the @ operator,

ePathKEGG_results <- ePathKEGG@result # You can access other slots the same way. For example, *ePathKEGG@params* reveals the parameters used in the analysis. Please note that some slots of the *ePathKEGG* S4 object are empty; for example, *gene2symbol* and *termsim* slots. The *result* slot is the most relevant and important slot.

To review the results in *Data Viewer* pane (upper-left pane), run the code below,

View(ePathKEGG_results) # Or, you just click the *ePatheKEGG_results* object in the *Environment* pane. You can now scroll up and down, left and right to review the results. You can also sort a specific column by clicking its column name.

To save the results on your hard drive in the CSV format,

write.csv(ePathKEGG_results, file = “KEGGpathwayEnrichment.csv”) # This saves the file in the working directory. You can write it into any folder when you provide the full path. You can open the saved file in Excel to review and process later.

### 9.3. Visualization of the KEGG Pathway Enrichment Results

#### 9.3.1. Dot Plots

Similar to visualization of GO enrichment, you can use *dotplot()* function to visualize the top 10 enriched pathways by default,

dotplot(ePathKEGG) # Generate dot plot using the default settings for the top 10 enriched pathways without distinguishing the activated and suppressed pathways.

You can customize the dot plot with arguments and *ggplot2* layer functions,

dotplot(ePathKEGG, title = “KEGG pathway enrichment”, split= “.sign”, label_format = 60) + facet_grid(“.~.sign”) + theme(axis.text.y = element_text(size = 11, face = “bold”))

**Figure 17:**
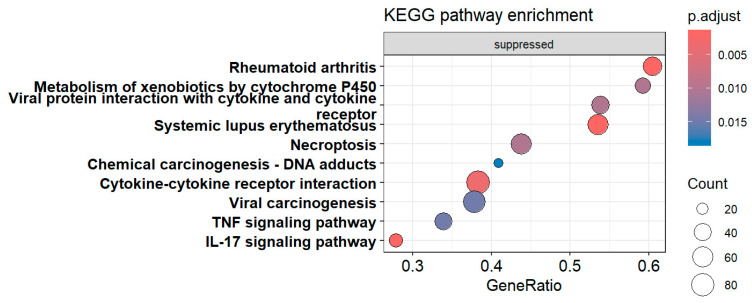
Dot plots for enriched KEGG pathways with split panels. Only one panel for the suppressed pathways can be seen, since no pathway is significantly activated.

The above code splits the activated and suppressed KEGG pathways and reveals that only suppressed pathways are enriched (Figure 17). We also modify the font sizes (from default 12 to 11) and face (to bold).

#### 9.3.2. Ridge Plots

ridgeplot(ePathKEGG) # This visualizes the top 30 enriched term by default.

#### 9.3.3. GSEA Plot

gseaplot(ePathKEGG, title = ePathKEGG@result$Description[1], geneSetID = 1)

The above code visualizes the top 1 enriched term with the term as the title of the plot. The title argument is defined by the code of ePathKEGG@result$Description[1].

#### 9.3.4. Term-Gene Network Plot

##### Generate the Term-Gene Network with ENTREZID as Gene Labels

cnetplot(ePathKEGG) # This generates a pathway-gene network plot, with genes labeled with the ENTREZID.

##### Generate Term-Gene Network with Human-Readable Gene Symbols

Genes in the above network plot are labeled with ENTREZID, and humans cannot readily understand those labels. We can use the *setReadable()* function from the DOSE package to include gene symbols in the S4 enrichment object,

ePathKEGGreadable <- setReadable(ePathKEGG, OrgDb = org.Hs.eg.db, keyType = “ENTREZID”)

Compare the *core_enrichment* columns of the two S4 objects and you can see that one is ENTREZID and the other has gene symbols. For example,

ePathKEGG$core_enrichment[1] # This prints out the entrez gene IDs of the first enriched term.

ePathKEGGreadable$core_enrichment[1] # This prints out the gene symbols for the first enriched term.

The above two codes treat *clusterProfiler* S4 object like a data frame and use extraction operator ($ and [ ]) to retrieve gene IDs (entrez ID and gene symbols).

Now, generate the term-gene network plot with the new S4 enrichment object,

cnetplot(ePathKEGGreadable) # The gene labels on the plot are now human-readable.

You can customize the term-gene network plot; for example,

cnetplot(ePathKEGGreadable, showCategory = 3, color_category = “black”, color_edge = “category”, foldChange = geneList4KEGGu)

To experience what happens with each added argument in the above code, you can add each argument one by one and run the new code from added *showCategory* to *foldChange* arguments. 

Colors are a key component of data visualization. You can find out all colors available in R using the command below,

colors() # This command prints out, in the RStudio *Console* pane, 672 colors available in R.

## 10. Anticipated Results

This tutorial will enable bench scientists to proficiently conduct FunCEA after practicing the procedures presented here. The tutorial had been tested by a sophomore college student and a lab scientist with a bachelor’s degree in biology. Both learned the skills within one week without much disruption of normal work and life. Both testers have very limited knowledge about R prior testing. Most codes in this tutorial take no time to execute and the audience can see results instantly. The enrichment analysis step (e.g., *enrichGO()* analysis) takes longer, but can be completed within one minute; for example, *enrichGO()* returns the results of *egoBP_down* in 15.6 s. Reproducibility is critical in science. With the same input gene list, the outcomes may be different due to use of different packages, various versions of packages, and releases of databases. It is advised that you run a *sessioninfo()* command to document tools used. The R *sessioninfo()* function can output information of the system and packages used in your analysis.

## 11. Limitation

Of note, a high-quality statistical process of the HTS data is essential for generation of a reliable input gene list for FunCEA. Users of this tutorial should be aware of advantage and disadvantages of different upstream tools, and choose the best statistics for your data analysis [16]. This tutorial focuses on FunCEA, and the upstream processes are out of the scope of this tutorial.

FunCEA relies on high-quality knowledgebases. Functional annotation is a dynamic process. Our current biological knowledgebases are far from completion and perfection. Software for FunCEA should use the most updated databases, and the same list of input genes may have different results when analyzed at different times.

## Figures and Tables

**Figure A1:**
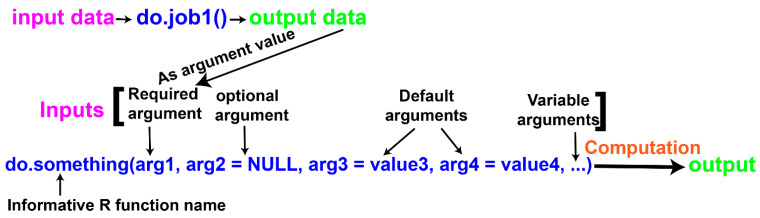
An R function is a mini-software. An R function (in blue text) receives data and parameters via function arguments, which is contained in parentheses, and conducts the designated task/computation. The nature of the task can usually be known by its function name. Conveniently, the output data of an R function (the upper one, *do.job1()*) can be used as the input values for an argument of another R function (the lower function in the figure). Therefore, it is critical to know what an R function does, and what data are needed and how to choose the parameters via arguments. For general users, it is not necessary to know the underlying codes that define the computation processes. Green text indicates output of an R function; purple text indicates input data for an R function to process; blue text indicates R functions.

**Figure A2:**
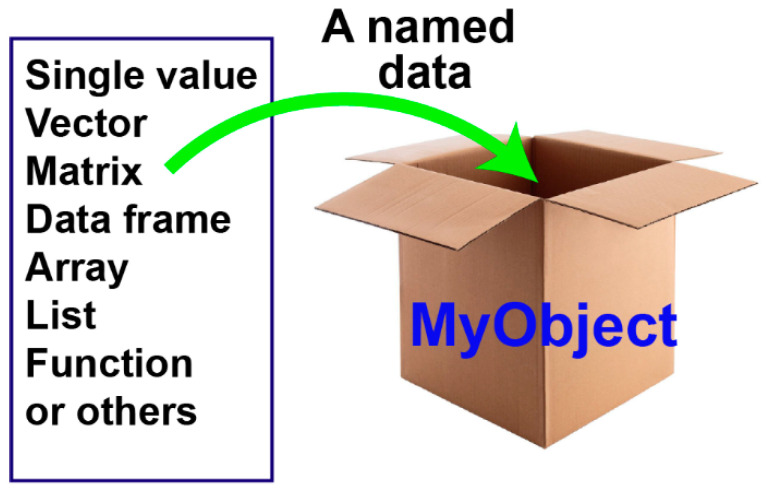
An R object is like a data container. The named data are temporarily stored in active memory and provide a convenient way for data processing and computation via the object name.

## Data Availability

No new data were created or analyzed in this study.

## Supplementary Materials

The following supporting information can be downloaded at: https://www.mdpi.com/article/10.3390/mps9010028/s1, R_Tutorial.*Rmd*, R_Tutorial.*html* and *OE_vs_KO.csv*.

## Abbreviations

BP	Biological process
CC	Cellular component
Cnetplot	Category network plot
CSV	Comma-separated values
FunCEA	Functional class enrichment analysis
FCS	Functional class scoring
GO	Gene ontology
GSEA	Gene set enrichment analysis
KEGG	Kyoto Encyclopedia of Genes and Genomes
MF	Molecular Function
ORA	Over-representation analysis
WD	Working directory

## Appendix A

### Appendix A.1. R Functions

For bench scientists to use R in data analysis and visualization, it is critical to understand R functions and how those work. Each R function can be understood as a miniature piece of software to implement a specific computation process or task. R function names are generally very informative and one knows what an R function does simply by its name alone. For example, *install.packages()* for installing R packages; *library()* for loading R packages from your package library; *mean()* to calculate means; *data.frame()* to generate an R data frame; *boxplot()* to calculate and generate box plots, and others. Like Word and PowerPoint applications, one does not need to know the underlying code of R functions to efficiently use them. Each R function automatically, precisely, and efficiently implements a task. Following the informative name of an R function are parentheses. Inside parentheses, there are one or more arguments. Arguments are parameters passed to an R function to control its operation. Each argument has a name and can receive user-provided data or pre-defined values. Users can define the arguments by providing data or pre-defined values to control how the computation process is done. There are different kind of arguments based on the nature of their values. R function arguments can have default values. NULL could be the default value, which means absent or undefined value, as a placeholder. When an argument has no default value, it is called a required argument, meaning that you need to provide values for the required argument for the function to work.

One can just run an R function by providing values for the required argument only without providing values to all arguments that do have default values. Sometimes, you can run an empty R function without providing any values; for example, when you run *date()*, it will output the current day, date, time, and year. When you simply execute *data()*, all built-in data sets are listed in the *Data Viewer* pane. When you issue *list.files()* without any arguments, it lists all visible files in the current working directory. Unlike object assignment operator (*<-*), you use the *=* assignment operator to specify values/data to an R argument. Argument names can be omitted only when you provide values/data in the order of the predefined order of the arguments inside the parentheses (refer to Section 5.3). The order of arguments does not matter when you include argument names in an R function. Figure A1 illustrates components of an R function and how it works.

The values/data of an argument of an R function can be generated by another function (i.e., the nested or internal function). Such use of R functions inside a function provides a convenient way for data processing. For example,

barplot(colMeans(USArrests)[c(1,2, 4)]) # This code includes three different functions to generate a bar plot using raw data from the built-in data set of *USArrests*.

The above code calculates means of each column on the built-in data set of *USArrests* using the *colMeans()* function inside the *barplot()* function. *colMeans()* generates a vector of four mean values. Then, it subsets mean values for *Murder*, *Assault*, and *Rape* per 10,000 people using the combine *c()* function and extraction operator *[ ]*. *c()* function generates a vector of indices for *[ ]* operator to subset the corresponding mean values. Finally, it generates bar plots from the resulting vector of three mean arrest rates in the USA. The above code can be broken into,

means <- colMeans(USArrests) # This script calculates means of each column using the *colMeans()* function and assigns the results to an R object *means*. For a description of R objects, refer to Appendix A.4. To see the output of this code, run means, or execute print(means).

indices <- c(1,2, 4) # It generates a vector of indices using the *c()* function, and puts this vector into the *indices* object. To see the output of the *c()* function here, you simply run indices, or execute print(indices).

data4plot <- means[indices] # This subsets mean values for *murder*, *assault*, and *rape* in the USA, and saves it into the *data4plot* object. The result of this subsetting can be seen by executing print(data4plot), or just data4plot.

barplot(data4plot) # The above four steps generate the same plot as the single code above using the nested functions. The plot appears in the *Plots* pane of RStudio.

The following is syntax for one of the visualization functions introduced in this tutorial, and description for each of its arguments.

dotplot(object, x = “GeneRatio”, color = “p.adjust”, showCategory = 10, size = NULL, split = NULL, font.size = 12, title = ““, orderBy = “x”, label_format = 30, ...)

#### Description of the enrichplot::dotplot() Function

**dotplot**: It is the name of this function. From this simple name, users know it calculates and generates dot plots. In fact, because it is a function from the *enrichplot* package, the full name for this function should be *enrichplot::dotplot()* to distinguish the same named function from the R base package *graphics* and the generic *lattice* package. However, *dotplot()* here implements *enrichplot::dotplot()* when *clusterProfiler* or *enrichplot* is loaded. When any of the latter two are loaded, the more generic *dotplot()* function from *graphics* or *lattice* is masked.

**( )**: The parentheses enclose the arguments or parameters.

**object**: This is the required argument. It is the only required input for *dotplot()* to generate a dot plot. The *object* argument of *enrichplot dotplot()* is an S4 enrichment object.

**x** argument defines values for the x-axis of the plot. It defaults to “*GeneRatio*”.

**color** defines what values are visualized by color. Its default is “*p.adjust*” values.

**showCategory**: It defines how many enriched terms are visualized. The top 10 enriched terms are visualized by default. It also accepts a vector of terms/category for visualization (see examples in the main tutorial).

**size** defines which statistical data are visualized by the dot sizes. It has a value of NULL. Although it defaults to NULL, *dotplot()* internally sets it to “*count*” or “*GeneRatio*” depending on x arguments to ensure both are visualized. Since x has a default of “*GeneRatio*”, size internally uses “*count*” as its default.

**split:** This argument allows for visualizing different enrichment categories in separate plot panels; for example, it allows CC, BP, and MF to be presented in three separate panels using the *ONTOLOGY* column in the ORA result data frame (see examples in this tutorial). NULL puts this argument as a placeholder. It does nothing by default.

**font.size** defines the font size of the x- and y-axis labels, but not that of the legend. The default is 12.

**title**: A plot title can be defined by this argument. The default is empty string, i.e., without a plot title.

**orderBy** defines how dots are ordered along the y-axis. The default value is “x”. The x-axis uses “*GeneRatio*”. Therefore, terms are ordered along the y-axis based on the values of “*GeneRatio*”.

**label_format** defines the maximum characters each line can accommodate for the y-axis labels. The default is 30 characters. If a term is longer than 30 characters, the 31st character will start a new line.

**…**: The three dots here mean that the users can add additional arguments.

### Appendix A.2. R Data Frame and Matrix

R data frames and matrices are each one of the basic R data structures. Both data frames and matrices are two-dimensional, looking like a rectangular common table. Each row is one observation; each column represents one feature of the data. All data in a matrix are of the same type: numeric, character, or logic, not a mix. In contrast, a data frame can have different types of data among its columns. However, each column of a data frame needs to be of the same type of data. R includes some built-in data frames and matrices; for example, *VADeaths* (a matrix) and *ToothGrowth* (a data frame). You can test whether those are matrices or data frames,

is.data.frame(VADeaths) # The answer is *FALSE* as you can see from return of this code.

is.data.frame(ToothGrowth) # The return is *TRUE*.

is.matrix(VADeaths) # The output is *TRUE*.

is.matrix(ToothGrowth) # You see a return of *FALSE*.

Data frames and matrices share many common functions for their manipulation, such as *nrow()*, *ncol()*, *dim()*, *summary()*, *head()*, *subset()*, and others. For example,

head(VADeaths) # This prints out the first 6 rows of the *VADeaths* matrix.

head(ToothGrowth) # This also prints out the first 6 rows of the *ToothGrowth* data frame.

We can access/extract a specific column of a data frame or a matrix using the extraction operator *$* or *[ ]*. Each element of a data frame or matrix can be located by its index, i.e., row and column number. For example, [ 3, 5] indicate the data point at row 3 and column 5. Using either $ or *[ ]*, we can generate a gene list for FunCEA from an expression data frame as shown in this tutorial. Here, we use *ToothGrowth* as an example to demonstrate how to extract one column and generate an R vector,

toothLength <- ToothGrowth$len # Extract the tooth length vector with the *$* operator by its column name, “len”.

toothLength2 <- ToothGrowth[, “len”] # Extract it by column name, i.e., “len” and the *[ ]* operator. The comma "," here means all rows are extracted for the “len” column.

toothLength3 <- ToothGrowth[, 1] # Extract tooth length vector by its index and the *[ ]* operator.

is.vector(toothLength) # The other two vectors can be tested similarly as shown here.

identical(toothLength, toothLength2) # You can test for the *toothLength* and *toothLength3* vectors similarly. The answer is *TRUE*, indicating the three methods generate the same result.

print(toothLength) # This prints out the vector. You can just run *toothLength* to print.

In this tutorial, the starting data of RNA-seq expression study are a data frame. The *result* slot of the enrichment S4 object is a data frame.

### Appendix A.3. R Vectors

An R vector is a list of data, in plain language. It is a one-dimensional data structure. Each item/data unit in a vector is called an R vector element. The elements of one R vector should be of the same type; for example, numeric or character, but not a mix of both. You use the combine function *c()* to generate an R vector. For example,

numbers <- c(1, 4, 6, 10) # This code generates a vector of four numbers and assigns it to the *numbers* object (refer to Appendix A.4 for R objects) using the *<-* assignment operator.

students <- c(“James”, “David”, “John”, “Smith”) # This script generates a vector of student names, a character vector, and assigns it to the *students* R object.

Each vector element can be named. For example,

geneExpression <- c(MYC = 10, OCT4 = 50, SOX2 = 30, KLF4 = 25) # This generates a named vector of gene expression. We use the assignment operator *=* to assign values to corresponding names of an R vector.

geneExpression # This outputs the vector values, i.e., the expression levels, along with corresponding names.

names(geneExpression) # This outputs the corresponding names of the vector’s elements.

Vector elements can be accessed or extracted by indices or names. For example,

geneExpression[“MYC”] # This code retrieves the expression level of MYC by the vector element name.

geneExpression [1] # This also retrieves the expression level of MYC, but by its index/location in the vector.

geneExpression[ c(“MYC”, “SOX2”)] # This script retrieves expression levels of MYC and SOX2 by a vector of names, i.e., *c(“MYC”, “SOX2”)*.

geneExpression[c(1, 3)] # This also extracts the expression levels of MYC and SOX2, but by a vector of indices/locations, i.e., *c(1,3)*.

In this tutorial, the input gene list is a vector. For FCS analysis, the input gene list should be a named vector.

### Appendix A.4. R Objects

Object is one of the fundamental concepts of R. There is a well-known saying about R objects: “everything that exists is an object”. R objects are containers that can hold data (a string, a vector, a data frame, a list, an array, and an S4 object), a function, or computation results (Figure A2). You should give an object a name. An R object is temporarily stored in the active memory of your computer. This named object allows one to use or compute on the object conveniently. R object names can use numbers and letters, but should not start with any number. Special characters should be avoided, but underscore and dot are generally used in object names. No space is allowed in an R object name. R is case-sensitive, and an R object of *genes* and *Genes* represent different R objects. R object names should be informative, and therefore phrases are frequently used. To make R objects more readable, you can use an underscore or a dot to separate different words; for example, *gene_list* or *gene.list*. Another practice is to use camel cases like *GeneList* and *sigUpData*, with the latter standing for “significantly up-regulated genes data set”. This tutorial uses a combination of underscore and camel case to name R objects. You use an assignment operator to store “anything” in an R object. The widely used assignment operator is the leftward assignment operator *<-*, but others like *-> *(rightward) and *=* (leftward) are used sometimes. For example,

geneList <- c(“MYC”, “SOX2”, “KLF4”, “OCT4”, “NANOG”) # This uses the leftward assignment operator *<-* to generate an R object of gene list vector.

c(“MYC”, “SOX2”, “KLF4”, “OCT4”, “NANOG”) -> gene.list # This uses the rightward assignment operator *->* to do this same thing as the first code does here.

geneList # This object allows you to print and review the gene list easily.

gene.list # Named objects like this allow one to print and review its content readily. In this case, the content is a list of genes.

You can even assign a function to a new object name. For example,

Dates <- date() # Assign the *date()* function to *Dates*.

date() # Run the *date()* function to display the results in the RStudio *Console* pane.

Dates # This object allows one to print out the date information as *date()* does.

Let us assign the built-in data frame of *iris* to a new name,

iris # This prints out the built-in data frame.

IRIS <- iris # This assigns *iris* to *IRIS*.

IRIS # Print out IRIS data frame to review.

identical(IRIS, iris) # The resulting objects allow you to compare them quickly.

You may generate many objects in a single project just like this tutorial. Those objects are gone if you do not save them onto your hard drive. You can save all of the objects (a workspace in R terminology) upon quitting your R session. To quit your R session, execute,

q()

RStudio will ask: “*Save workspace image to ~/.RData? [y/n]:*”. All generated objects will be saved in the hidden file of “.RData” if you answer it with a “y”. This file is saved in your current working directory.

You can save the workspace in any directory with an informative file name using the *save.image()* function. For example,

save.image(file = “FunCEA_tutorial.RData”) # This saves the workspace on the working directory. You can save it in any other directory when the path is provided.

When you come back and continue any unfinished work, you can load the workspace using the *load()* function to retrieve all of the objects back to R,

load(file = “FunCEA_tutorial.RData”) # After executing this script, you will see all of the objects generated previously appear in the *Environment* pane.

**Table A1:** Troubleshooting table.

Warning Message/Issues	Cause	Solutions
Error: unexpected input in “rawData <- read.csv(““	Curly quotation marks used due to direct copy of code from Word or PowerPoint	Use straight quotation marks in R
Unused argument	Misspelling; no such an argument	R is case sensitive, check spelling
Function not found	Misspelling; package not loaded	Check spelling; load package
*geom_cnet_label() not found*	ggtangle package not loaded when using cnetplot()	Load the ggtangle library
Could not find function “theme”	ggplot2 not loaded	Load ggplot2
Could not find function “element.text”	Tidyvesre use underscore in its function names, not dot	Use element_text()
See “no ‘dimnames’ attribute for array” when use emapplot()	Similarity matrix is missing	Generate similarity matrix using pairwise_termsim()
Could not find function “pairwise_termsim”	enrichplot not loaded	Load enrichplot package
Could not find function “%>%”	Package dplyr not loaded	Load dplyr
Object ‘org.Hs.eg.db’ not found	org.Hs.eg.db not loaded	Load org.Hs.eg.db
geneList should be a decreasing sorted vector	Gene list for FCS is not ranked	Rank the gene list with sort()
Could not find function “mapIds”	Package AnnotationDbi not loaded	Load AnnotationDbi or org.Hs.eg.db
Argument “keytype” is missing; or “unused argument keytype”	Likely typo; R is case sensitive	Use keyType instead
Error in x =“geneRatio”	Likely you do not use argument name for dotplot(), for example showCategory is missing	Add *showCategory =* in dotplot() when you do not use its default
