# Supplementary material for: Biological Functional Class Enrichment Analysis with R, an Annotated Tutorial for Bench Scientists
Source: Methods Protoc. 2026 Feb 19;9(1):28. doi: 10.3390/mps9010028 (PMC12943126; doi:10.3390/mps9010028)
Supplement: Supplementary file 1 [file mps-09-00028-s001.zip › supplemental/R_Tutorial.html]

R\_Tutorial.knit


# Term Enrichment tutorial

#### Kejin Hu

#### 2026-02-06

# Introduction

This is the R Markdown version of “Biological functional class
enrichment analysis with R, an annotated tutorial for bench scientists”.
The R scripts in this version include the major steps but some
supporting codes may be omitted. ggplot2 grammars are used for most
plots in the R Markdown version for include of plot title and
customization of title text. Audience can ignore the ggplot2 parts of
the code and focus on the use of plotting functions when You do not feel
comfortable with ggplot2. Those added plot titles are intented to help
audience better understand the plots. The section headings may not be
the same as the tutorial and some sections are not included here, for
example, sections of package installation and section of Materials and
Equipment. This file was added as suggested by one reviewer, who found
that copying scripts from the PDF tutorial frequently caused issues due
to different text or character coding systems (PDF, Word vs R). The R
Markdown version allows the audience to test the codes in R without
typing. Comments on R scripts are indicated by a # following or before
the script. The code chunk is highlighted by pale pink background. The
output of the code is in soft lemon background. For best outcome,render
this R Markdown file to HTML, not PDF nor Word.

# Load the required R packages.

The following external R packages are required for this tutorial.
*clusterProfiler* provides many enrichment analysis functions.
*org.Hs.eg.db* is the annotation package for human genes.
*ReactomePA* provides enrichment functions for pathway analysis
using the reactome database. *enrichplot* package has many
functions for visualization of enrichment results. *ggplot2*
includes tools for plot customization of non-data elements of plots, and
*dplyr* provides some functions for data processing. Some other
packages are essential but do not need being loaded explicitly, for
example, *GO.db* and *AnnotationDbi*.

```
library(clusterProfiler)
library(org.Hs.eg.db)
library(enrichplot)
library(ReactomePA)
library(ggplot2)
library(dplyr)
```

# ORA for GO term enrichment and visualization

## Preparation of input gene list from raw data

First, we input the raw expression data into R from the folder of
*classEnrichment*, using the base R function
*read.csv()*.

```
setwd("C:/Users/kh0794/Documents/classEnrichment")  # Set up the folder containing your original expression data as the working directory. Your path is different from what you see here.

rawData <- read.csv("OE_vs_KO.csv") # It reads in the file "OE_vs_KO.csv" from the working directory, and stores it in the rawData object.

dim(rawData) # Find out the dimension of rawData data frame, i.e., numbers of rows and columns.
```

```
## [1] 35953    14
```

Subset the data for the differentially expressed genes (down and up)
using the base R function *subset()*.

```
sigDownData <- subset(rawData, padj < 0.05 & log2FoldChange < -1) # This extracts the subset of down-regulated genes at padj < 0.05 level and 2-fold.

sigUpdata <- subset(rawData, log2FoldChange > 1 & padj < 0.05)
```

Generate input gene list for enrichment analysis. This tutorial uses
the list of down-regulated genes as an example. For the upregulated
genes, similar procedures can be used.

```
sigDownGeneList <- sigDownData$log2FoldChange # Generate a list with log2FoldChange values.

names(sigDownGeneList) <- sigDownData$ENSEMBL # Name each log2FoldChange with its corresponding ENSEMBL ID.

head(sigDownGeneList)   # Print out the first 6 of the gene list.
```

```
## ENSG00000162415 ENSG00000117525 ENSG00000183508 ENSG00000143631 ENSG00000234810 
##       -1.160773       -2.896303       -1.579464       -2.811414       -1.452279 
## ENSG00000092969 
##       -1.217003
```

```
head(names(sigDownGeneList))  # List the first 6 ENSEMBLE IDs using the names() function.
```

```
## [1] "ENSG00000162415" "ENSG00000117525" "ENSG00000183508" "ENSG00000143631"
## [5] "ENSG00000234810" "ENSG00000092969"
```

## GO enrichment analysis using *enrichGO()* and GO database

### Conduct the enrichment analysis and overview the results

#### Prepare the enrichment S4 object

The following script generates the enrichment results for biological
process (BP) of gene ontology (GO) using the *enrichGO()*
function from *clusterProfiler*. It stores the results in an S4
object, egoBP. Here, we demonstrate with the down-regulated genes only.
The up-regulated genes can be analyzed similarly. Unlike the first
enrichment analysis in the main tutorial, this script below directly
generates results with human readable gene symbols (*readable =
TRUE*) so that we can generate term-gene network plots with gene
symbols rather than ENSEMBL IDs, which humans cannot readily
understand.

```
egoBP_down <- enrichGO(names(sigDownGeneList), OrgDb = org.Hs.eg.db, keyType = "ENSEMBL", ont = "BP", readable = T)
```

#### Overview the results.

The following simple command can print out the summary of the
enrichment results including type of enrichment analysis, parameters
used, number of enriched terms, and a truncated data frame of the
enrichment results, as well as the citation of the
*cluterProfiler* package.

```
egoBP_down # This is implicit use of print(egoBP_down).
```

```
## #
## # over-representation test
## #
## #...@organism     Homo sapiens 
## #...@ontology     BP 
## #...@keytype      ENSEMBL 
## #...@gene     chr [1:602] "ENSG00000162415" "ENSG00000117525" "ENSG00000183508" ...
## #...pvalues adjusted by 'BH' with cutoff <0.05 
## #...551 enriched terms found
## 'data.frame':    551 obs. of  12 variables:
##  $ ID            : chr  "GO:0033002" "GO:0048659" "GO:0048660" "GO:0030198" ...
##  $ Description   : chr  "muscle cell proliferation" "smooth muscle cell proliferation" "regulation of smooth muscle cell proliferation" "extracellular matrix organization" ...
##  $ GeneRatio     : chr  "30/403" "24/403" "23/403" "28/403" ...
##  $ BgRatio       : chr  "273/21273" "199/21273" "195/21273" "394/21273" ...
##  $ RichFactor    : num  0.1099 0.1206 0.1179 0.0711 0.0709 ...
##  $ FoldEnrichment: num  5.8 6.37 6.23 3.75 3.74 ...
##  $ zScore        : num  11.09 10.57 10.19 7.66 7.64 ...
##  $ pvalue        : num  9.57e-15 6.30e-13 3.08e-12 2.40e-09 2.54e-09 ...
##  $ p.adjust      : num  4.20e-11 1.38e-09 4.50e-09 2.07e-06 2.07e-06 ...
##  $ qvalue        : num  3.21e-11 1.06e-09 3.44e-09 1.58e-06 1.58e-06 ...
##  $ geneID        : chr  "TGFB2/PTGS2/TACR1/CALCRL/NPPC/PDE1A/ADIPOQ/IL12A/APOD/HPGD/EREG/MEF2C/JARID2/HTR1B/SOD2/TNFAIP3/MYB/HGF/IL6/MIR"| __truncated__ "PTGS2/TACR1/CALCRL/NPPC/PDE1A/ADIPOQ/IL12A/APOD/HPGD/EREG/MEF2C/HTR1B/SOD2/TNFAIP3/MYB/IL6/MIR221/APLN/CCN3/CCN"| __truncated__ "PTGS2/TACR1/CALCRL/NPPC/PDE1A/ADIPOQ/IL12A/APOD/HPGD/EREG/MEF2C/HTR1B/SOD2/TNFAIP3/MYB/IL6/MIR221/APLN/CCN4/NR4"| __truncated__ "TGFB2/ADAMTS4/ELF3/COL11A1/COL5A2/CYP1B1/COL3A1/PTX3/CCDC80/SLC39A8/COL11A2/IL6/SULF1/MMP16/TNFRSF11B/HAS2/MMP1"| __truncated__ ...
##  $ Count         : int  30 24 23 28 28 28 17 6 29 28 ...
## #...Citation
## S Xu, E Hu, Y Cai, Z Xie, X Luo, L Zhan, W Tang, Q Wang, B Liu, R Wang, W Xie, T Wu, L Xie, G Yu. Using clusterProfiler to characterize multiomics data. Nature Protocols. 2024, 19(11):3292-3320
```

egoBP\_down is an S4 object. The primary components of an S4 object is
slots. Each slot holds a specific type of result information. For
example, the first slot, *result* holds the result data frame.
The *universe* slot holds the reference genes. Its slot names can
be listed with the *slotNames()* function,

```
slotNames(egoBP_down)
```

```
##  [1] "result"        "pvalueCutoff"  "pAdjustMethod" "qvalueCutoff" 
##  [5] "organism"      "ontology"      "gene"          "keytype"      
##  [9] "universe"      "gene2Symbol"   "geneSets"      "readable"     
## [13] "termsim"       "method"        "dr"
```

Generate data frame for the entire statistical data from the
*result* slot, using the @ operator. Then, we print out the
column names of the resulting data frame, its dimension, and list it
first row.

```
egoBP_downRes <- egoBP_down@result

names(egoBP_downRes)  # This prints out the column names of the results data frame.
```

```
##  [1] "ID"             "Description"    "GeneRatio"      "BgRatio"       
##  [5] "RichFactor"     "FoldEnrichment" "zScore"         "pvalue"        
##  [9] "p.adjust"       "qvalue"         "geneID"         "Count"
```

```
dim(egoBP_downRes)  # Find out how many terms (rows) are there.
```

```
## [1] 4386   12
```

```
head(egoBP_downRes, n = 1)  # This print out the first row of the resulting data frame.
```

```
##                    ID               Description GeneRatio   BgRatio RichFactor
## GO:0033002 GO:0033002 muscle cell proliferation    30/403 273/21273  0.1098901
##            FoldEnrichment   zScore       pvalue     p.adjust       qvalue
## GO:0033002       5.800725 11.09364 9.571603e-15 4.198105e-11 3.205983e-11
##                                                                                                                                                                               geneID
## GO:0033002 TGFB2/PTGS2/TACR1/CALCRL/NPPC/PDE1A/ADIPOQ/IL12A/APOD/HPGD/EREG/MEF2C/JARID2/HTR1B/SOD2/TNFAIP3/MYB/HGF/IL6/MIR221/APLN/CCN3/CCN4/ANGPT1/NRG1/NR4A3/FOS/BMP4/BMP2/ADAMTS1
##            Count
## GO:0033002    30
```

#### Generate data frame for the enriched terms only.

Using the @ operator above, we extract data frame of statistical data
for all terms analyzed including enriched and non-enriched. The
following method extracts a statistical data frame for enriched terms
only (excluding the non-enriched terms).

```
egoBP_downEnrichedRes <- as.data.frame(egoBP_down)

dim(egoBP_downEnrichedRes)  # This shows that many less rows are in this data frame as compared to egoBP_downRes because the former is enriched terms while the latter is all terms analyzed.
```

```
## [1] 551  12
```

```
head(egoBP_downEnrichedRes, n = 1)  # This lists statistical data for the first enriched term, i.e., the first row of the data frame.
```

```
##                    ID               Description GeneRatio   BgRatio RichFactor
## GO:0033002 GO:0033002 muscle cell proliferation    30/403 273/21273  0.1098901
##            FoldEnrichment   zScore       pvalue     p.adjust       qvalue
## GO:0033002       5.800725 11.09364 9.571603e-15 4.198105e-11 3.205983e-11
##                                                                                                                                                                               geneID
## GO:0033002 TGFB2/PTGS2/TACR1/CALCRL/NPPC/PDE1A/ADIPOQ/IL12A/APOD/HPGD/EREG/MEF2C/JARID2/HTR1B/SOD2/TNFAIP3/MYB/HGF/IL6/MIR221/APLN/CCN3/CCN4/ANGPT1/NRG1/NR4A3/FOS/BMP4/BMP2/ADAMTS1
##            Count
## GO:0033002    30
```

You can subset enriched term data directly from the S4 results object
using the traditional *[ ]* extraction operator for data
frame.

```
top10term <- egoBP_down[1:10] # This extract the top 10 enriched BP terms.
```

### Remove the redundant terms using the *simplify()* function.

Many enriched terms are redundant and *clusterProfiler*
provides the *simplify()* function to remove the redundant
terms.

```
egoBP_downSim <- clusterProfiler::simplify(egoBP_down) # We use cluserProfiler::simplify() rather than simplify() to avoid namespace conflict. 

dim(egoBP_downEnrichedRes)
```

```
## [1] 551  12
```

```
dim(egoBP_downSim)  # Check the enriched term directly on S4 object using dim() function.
```

```
## [1] 241  12
```

```
isS4(egoBP_downSim) # Test if it is an S4 object.
```

```
## [1] TRUE
```

```
egoBP_downSimRes <- egoBP_downSim@result  # Generate result data frame from S4 object using the @ operator 

egoBP_downSimEnrichEd <- as.data.frame(egoBP_downSim)

identical(egoBP_downSimRes, egoBP_downSimEnrichEd)  # As you can see the two data frames are the same since the simplified S4 result object includes enriched terms only.
```

```
## [1] TRUE
```

### Extract the experimental gene list from an enriched term

#### Extract the subset of a term gene set as it is in the data frame, i.e., one string.

```
termGeneString1 <- egoBP_downSim[4]["geneID"]  # This extracts values at the geneID column of the fourth row. It includes the gene symbols for the DEG subset of the 4th enriched term.

termGeneString1 # Print out its content.
```

```
##                                                                                              geneID
## GO:0045471 RGS2/RGS4/TACR1/NPPC/ADIPOQ/HPGD/CDO1/SLC6A3/HTR1B/SOD2/PENK/ARC/FOS/ABAT/NQO1/CCL7/FOSB
```

```
termGeneString2 <- egoBP_downSimRes[egoBP_downSimRes$Description == "response to ethanol" ,]["geneID"]  # This script extracts the DEG subset for the enriched term by the term name, which is in the Description column of the enrichment data frame. 

# Alternatively, you can use the following code,which will remain correct even though knowledgebased will change. 
termGeneString2b <- egoBP_downSimRes[egoBP_downSimRes$Description == egoBP_downSim[4]$Description ,]["geneID"]

identical(termGeneString1, termGeneString2b)
```

```
## [1] TRUE
```

```
termGeneString2 # Print out the one-column one-row data frame.
```

```
##                                                                                              geneID
## GO:0045471 RGS2/RGS4/TACR1/NPPC/ADIPOQ/HPGD/CDO1/SLC6A3/HTR1B/SOD2/PENK/ARC/FOS/ABAT/NQO1/CCL7/FOSB
```

```
identical(termGeneString1, termGeneString2)  # This tests if the two methods above result in the identical data. Please note that at the time of your practice, "response to ethanol" may not be the fourth term due to update of knowledgebase.
```

```
## [1] TRUE
```

```
is.data.frame(termGeneString1) # But, those are data frames as tested here. It is a data frame with one row and one column.You can also tell they are data frames from their printout in which there is a column name of "geneID" along with a row name of GO ID.
```

```
## [1] TRUE
```

```
termGeneString <- termGeneString1$geneID  # This extracts genes as a vector using the $ extract operator.

termGeneString # List the content of the vector. As you can see there is no names of row and column in this printout.
```

```
## [1] "RGS2/RGS4/TACR1/NPPC/ADIPOQ/HPGD/CDO1/SLC6A3/HTR1B/SOD2/PENK/ARC/FOS/ABAT/NQO1/CCL7/FOSB"
```

```
is.vector(termGeneString) # It is a vector as tested by is.vector().
```

```
## [1] TRUE
```

```
termGeneString3 <- egoBP_downSimRes[egoBP_downSimRes$Description == "response to ethanol" ,]$geneID  # This directly generates a vector of genes for the enriched term of "response to ethanol".

termGeneString3
```

```
## [1] "RGS2/RGS4/TACR1/NPPC/ADIPOQ/HPGD/CDO1/SLC6A3/HTR1B/SOD2/PENK/ARC/FOS/ABAT/NQO1/CCL7/FOSB"
```

```
identical(termGeneString, termGeneString3)   # The two vectors are the same.
```

```
## [1] TRUE
```

#### Convert genes in one-string vector into a vector of genes with separate individual genes

Separate genes of an enriched term using the *strsplit()*
function.

```
termGeneList <- strsplit(termGeneString3, split = "/") # This separates the genes and removes the separator /.
```

Check the length of the resulting list. It is 1 rather than number of
the differentially expressed genes because it is an R list with one
component only.

```
length(termGeneList)
```

```
## [1] 1
```

Print out the R list for this enriched term to see how an R list
looks like.

```
termGeneList
```

```
## [[1]]
##  [1] "RGS2"   "RGS4"   "TACR1"  "NPPC"   "ADIPOQ" "HPGD"   "CDO1"   "SLC6A3"
##  [9] "HTR1B"  "SOD2"   "PENK"   "ARC"    "FOS"    "ABAT"   "NQO1"   "CCL7"  
## [17] "FOSB"
```

Convert the R list into an R vector using the *unlist()*
function,

```
termGene_vector <- unlist(termGeneList)
```

Check the length of the resulting vector. You can see now the length
is the number of genes.

```
length(termGene_vector)
```

```
## [1] 17
```

Print out the genes for this enriched term to see different looks of
an R list and vector of the same group of genes.

```
termGene_vector # As you can see it is now a vector of gene.
```

```
##  [1] "RGS2"   "RGS4"   "TACR1"  "NPPC"   "ADIPOQ" "HPGD"   "CDO1"   "SLC6A3"
##  [9] "HTR1B"  "SOD2"   "PENK"   "ARC"    "FOS"    "ABAT"   "NQO1"   "CCL7"  
## [17] "FOSB"
```

### Generate a list of genes from a group of related enriched terms by index

The following codes extract the enriched terms with the string
pattern of “lipid”. We first find out such terms to review; then find
their indices; finally, extract the genes as one single vector with
their indices. Some genes are redundant (appear in multiple terms) in
the vector and we use the *unique()* function to obtain the
unique set of genes.

```
grep("lipid", egoBP_downSimRes$Description, value = TRUE) # This lists the enriched BP terms with "lipid" keyword.
```

```
## [1] "positive regulation of lipid transport"   
## [2] "positive regulation of lipid localization"
## [3] "lipid export from cell"                   
## [4] "lipid digestion"
```

```
grep("lipid", egoBP_downSimRes$Description)  # This lists the indices of the lipid BP terms. The indices will be used in the next step.
```

```
## [1]  46  53  56 140
```

```
lipidGeneList <- strsplit(egoBP_downSimRes$geneID[c(46, 53, 56, 140)], split = "/")

lipidGeneList # This prints out the resulting list of genes with roles in lipid biology. As you can see this list has four components corresponding to the four terms.
```

```
## [[1]]
##  [1] "PLA2G4A" "IL1B"    "IL1A"    "ADIPOQ"  "SPP1"    "MYB"     "NKX3-1" 
##  [8] "LPCAT3"  "ABCA5"   "LIPG"   
## 
## [[2]]
##  [1] "PLA2G4A" "IL1B"    "IL1A"    "ADIPOQ"  "SPP1"    "MYB"     "CD36"   
##  [8] "NKX3-1"  "LPCAT3"  "ABCA5"   "LIPG"   
## 
## [[3]]
## [1] "PLA2G4A" "PTGS2"   "IL1B"    "IL1A"    "SPP1"    "MYB"     "ACSL4"  
## [8] "NKX3-1" 
## 
## [[4]]
## [1] "CD36"   "AKR1C1" "LPCAT3" "LDLR"
```

```
lipidGenesVector <- unlist(lipidGeneList)  # This converts R list into an R vector. 

lipidGenesVector
```

```
##  [1] "PLA2G4A" "IL1B"    "IL1A"    "ADIPOQ"  "SPP1"    "MYB"     "NKX3-1" 
##  [8] "LPCAT3"  "ABCA5"   "LIPG"    "PLA2G4A" "IL1B"    "IL1A"    "ADIPOQ" 
## [15] "SPP1"    "MYB"     "CD36"    "NKX3-1"  "LPCAT3"  "ABCA5"   "LIPG"   
## [22] "PLA2G4A" "PTGS2"   "IL1B"    "IL1A"    "SPP1"    "MYB"     "ACSL4"  
## [29] "NKX3-1"  "CD36"    "AKR1C1"  "LPCAT3"  "LDLR"
```

```
lipidGenes_unique <- unique(lipidGenesVector) # This removes the redundant genes in the R vector. 

lipidGenes_unique # It prints out the genes. As you can see the list is shorter after removing the redundant ones.
```

```
##  [1] "PLA2G4A" "IL1B"    "IL1A"    "ADIPOQ"  "SPP1"    "MYB"     "NKX3-1" 
##  [8] "LPCAT3"  "ABCA5"   "LIPG"    "CD36"    "PTGS2"   "ACSL4"   "AKR1C1" 
## [15] "LDLR"
```

## Visualize the enrichment results

### Dot plot

The following three scripts all generate dot plots of the enrichment
results. The first one plots the top 5 enriched terms as defined by
*showCategory = 5*. The second one plots three selected enriched
terms also defined by the *showCategory* argument. The third one
plots enriched lipid-related terms. Each plot has a figure title added
by the ggplot2 function *ggtitle()*.

```
# Script below plots the top 5 enriched terms. 
dotplot(egoBP_downSim, showCategory = 5) + ggtitle("Dot plot for the top five enriched BP terms") + theme(plot.title = element_text(colour = "blue", face= "bold", hjust = 0.5), plot.title.position = "plot")
```

```
# The code below plots the three terms listed  in the showCategory argument. 
dotplot(egoBP_downSim, showCategory = c("response to ethanol", "muscle cell proliferation", "response to nutrient")) + ggtitle("Dot plot for the selected enriched BP terms") + theme(plot.title = element_text(colour = "blue", face= "bold", hjust = 0.5), plot.title.position = "plot")
```

```
 # This script below generates dot plots for enriched terms with string pattern of "lipid" or "stero", which include lipid, steroid, sterol, and cholesterol. 
dotplot(egoBP_downSim, showCategory = grep("lipid|stero", egoBP_downSim$Description, value = TRUE)) + ggtitle(" Dot plot for the enriched BP terms of lipogenesis") + theme(plot.title = element_text(colour = "blue", face= "bold", hjust = 0.5), plot.title.position = "plot")
```

### Visualize all three GO sub-categories in one figure as three separate dot plots.

```
egoALLdown <- enrichGO(gene = names(sigDownGeneList), OrgDb = org.Hs.eg.db, keyType = "ENSEMBL", ont = "ALL", readable = TRUE) # This script generates the enrichment S4 object for all 3 GO categories. 

egoALLdown # This command prints out the overview of the enrichment results.
```

```
## #
## # over-representation test
## #
## #...@organism     Homo sapiens 
## #...@ontology     GOALL 
## #...@keytype      ENSEMBL 
## #...@gene     chr [1:602] "ENSG00000162415" "ENSG00000117525" "ENSG00000183508" ...
## #...pvalues adjusted by 'BH' with cutoff <0.05 
## #...590 enriched terms found
## 'data.frame':    590 obs. of  13 variables:
##  $ ONTOLOGY      : chr  "BP" "BP" "BP" "BP" ...
##  $ ID            : chr  "GO:0033002" "GO:0048659" "GO:0048660" "GO:0030198" ...
##  $ Description   : chr  "muscle cell proliferation" "smooth muscle cell proliferation" "regulation of smooth muscle cell proliferation" "extracellular matrix organization" ...
##  $ GeneRatio     : chr  "30/403" "24/403" "23/403" "28/403" ...
##  $ BgRatio       : chr  "273/21273" "199/21273" "195/21273" "394/21273" ...
##  $ RichFactor    : num  0.1099 0.1206 0.1179 0.0711 0.0709 ...
##  $ FoldEnrichment: num  5.8 6.37 6.23 3.75 3.74 ...
##  $ zScore        : num  11.09 10.57 10.19 7.66 7.64 ...
##  $ pvalue        : num  9.57e-15 6.30e-13 3.08e-12 2.40e-09 2.54e-09 ...
##  $ p.adjust      : num  4.20e-11 1.38e-09 4.50e-09 2.07e-06 2.07e-06 ...
##  $ qvalue        : num  3.21e-11 1.06e-09 3.44e-09 1.58e-06 1.58e-06 ...
##  $ geneID        : chr  "TGFB2/PTGS2/TACR1/CALCRL/NPPC/PDE1A/ADIPOQ/IL12A/APOD/HPGD/EREG/MEF2C/JARID2/HTR1B/SOD2/TNFAIP3/MYB/HGF/IL6/MIR"| __truncated__ "PTGS2/TACR1/CALCRL/NPPC/PDE1A/ADIPOQ/IL12A/APOD/HPGD/EREG/MEF2C/HTR1B/SOD2/TNFAIP3/MYB/IL6/MIR221/APLN/CCN3/CCN"| __truncated__ "PTGS2/TACR1/CALCRL/NPPC/PDE1A/ADIPOQ/IL12A/APOD/HPGD/EREG/MEF2C/HTR1B/SOD2/TNFAIP3/MYB/IL6/MIR221/APLN/CCN4/NR4"| __truncated__ "TGFB2/ADAMTS4/ELF3/COL11A1/COL5A2/CYP1B1/COL3A1/PTX3/CCDC80/SLC39A8/COL11A2/IL6/SULF1/MMP16/TNFRSF11B/HAS2/MMP1"| __truncated__ ...
##  $ Count         : int  30 24 23 28 28 28 17 6 29 28 ...
## #...Citation
## S Xu, E Hu, Y Cai, Z Xie, X Luo, L Zhan, W Tang, Q Wang, B Liu, R Wang, W Xie, T Wu, L Xie, G Yu. Using clusterProfiler to characterize multiomics data. Nature Protocols. 2024, 19(11):3292-3320
```

```
egoALLdownSim <- clusterProfiler::simplify(egoALLdown) # This removes the redundant terms.

dotplot(egoALLdownSim, showCategory =7, split = "ONTOLOGY", font.size=10, label_format=23) + facet_grid(.~ONTOLOGY) + theme(panel.grid.major.y = element_line(color = "grey50")) + ggtitle("Top enriched GO terms for all three categories (BP, CC and MF)")  + theme(plot.title = element_text(colour = "blue", face = "bold", hjust = 0.5), plot.title.position = "plot")
```

### Visualize gene network of the enriched terms

#### Generate term-gene network for the top three enriched terms.

```
cnetplot(egoALLdownSim, showCategory = 3) + ggtitle("Term-gene network plot for enriched GO terms") + theme(plot.title = element_text(color = "blue", hjust = 0.5, face = "bold"), plot.title.position = "plot")
```

#### Generate the category-gene network for related enriched term names.

This plot also modifies the colors of edge, colors of nodes, and
visualizes the expression levels of member genes.This codes also makes
the line (edge) thicker. ggplot2 functions are used to add figure title
and modify the title text. The term vector in the showCategory argument
is selected using the *grep()* function.

```
cnetplot(egoALLdownSim, showCategory = grep("lipid|stero", egoALLdownSim$Description, value = T), color_edge = "category", foldChange = sigDownGeneList, color_category = "black", size_edge = 1) + ggtitle("Term-gene network plot for enriched terms in lipogenesis") + theme(plot.title = element_text(colour = "blue", face = "bold", hjust = 0.5))
```

### Generate enrichment maps among enriched terms.

#### Prepare the term similarity matrix frist

```
egoALLdownSim@termsim # Check if the term similarity matrix exists.
```

```
## <0 x 0 matrix>
```

```
egoBP_downSim <- pairwise_termsim(egoALLdownSim) # Generate term similarity matrix.

head(egoBP_downSim@termsim, n = 1)[1:10] # Review the first 10 values of the first row in the generated matrix. Please note, the entire matrix is big. Even one row will generate a lot of clutters.
```

```
##  [1] 1.00000000 0.76666667 0.07407407 0.17500000 0.05882353 0.18000000
##  [7] 0.18367347 0.20000000 0.06818182 0.12000000
```

```
emapplot(egoBP_downSim, showCategory = 20) + ggtitle("Enrichment map for the top 20 enriched BP terms") + theme(plot.title = element_text(hjust = 0.5, colour = "blue", face = "bold"))
```

# ORA pathway enrichment analysis

## Add entrez IDs to the original data frame as a matching column.

### Generate a data frame with entrez ID

```
anno <- AnnotationDbi::select(org.Hs.eg.db, keys = rawData$ENSEMBL, keytype = "ENSEMBL", columns = "ENTREZID") # Extract ENTREZID based on ENSEMBL keys from  rawData. 

head(anno) # See the first 6 rows of the resulting data frame.
```

```
##           ENSEMBL  ENTREZID
## 1 ENSG00000279928      <NA>
## 2 ENSG00000228037      <NA>
## 3 ENSG00000142611     63976
## 4 ENSG00000157911      5192
## 5 ENSG00000224340 100270877
## 6 ENSG00000142655      5195
```

```
dim(anno) # Find out the dimension of the anno data frame.
```

```
## [1] 36994     2
```

```
dim(rawData) # Print out the dimension of rawData to compare.
```

```
## [1] 35953    14
```

```
anno <- distinct(anno, ENSEMBL, .keep_all = TRUE) # Remove the duplicated ENSEMBL IDs.

dim(anno) # Check the dimension of the new anno data frame.
```

```
## [1] 35953     2
```

```
rawDataEntrez <- left_join(rawData, anno, by = "ENSEMBL") # Combine the two data frames into one. 

dim(rawDataEntrez) # Check the new data frame with ENTREZID as a column.
```

```
## [1] 35953    15
```

```
rawDataEntrez <- rawDataEntrez[!is.na(rawDataEntrez$ENTREZID),] # Remove the NA in the ENTREZID column. 

dim(rawDataEntrez) # Print out the dimension to see the difference between before and after NA removal.
```

```
## [1] 24111    15
```

### Generate the input gene list for pathway enrichment analysis

```
sigUpDataEnt <- subset(rawDataEntrez, log2FoldChange > 1 & padj < 0.05) # Subset data for the up-regulated genes. 

sigDownDataEnt <- subset(rawDataEntrez, log2FoldChange < -1 & padj < 0.05) # Subset dat for the down-regulated genes (2-fold change as the threshold.)

sigDownGeneListEnt <- sigDownDataEnt$log2FoldChange # Extract relative expression values for each down-regulated genes. 

names(sigDownGeneListEnt) <- sigDownDataEnt$ENTREZID # Names each log2FoldChange with its corresponding ENTREZID.

head(sigDownGeneListEnt) # List the first 6 genes to review.
```

```
##     57643      2152     54855      2312      7042      5321 
## -1.160773 -2.896303 -1.579464 -2.811414 -1.217003 -1.569461
```

### Conduct the pathway enrichment analysis and review the results.

```
ePathDown <- enrichPathway(names(sigDownGeneListEnt), readable = T)

ePathDown # Overview the enrichment results.
```

```
## #
## # over-representation test
## #
## #...@organism     human 
## #...@ontology     Reactome 
## #...@keytype      ENTREZID 
## #...@gene     chr [1:506] "57643" "2152" "54855" "2312" "7042" "5321" "8707" "102724548" ...
## #...pvalues adjusted by 'BH' with cutoff <0.05 
## #...87 enriched terms found
## 'data.frame':    87 obs. of  12 variables:
##  $ ID            : chr  "R-HSA-373076" "R-HSA-191273" "R-HSA-500792" "R-HSA-6783783" ...
##  $ Description   : chr  "Class A/1 (Rhodopsin-like receptors)" "Cholesterol biosynthesis" "GPCR ligand binding" "Interleukin-10 signaling" ...
##  $ GeneRatio     : chr  "25/294" "7/294" "29/294" "8/294" ...
##  $ BgRatio       : chr  "334/11214" "27/11214" "468/11214" "47/11214" ...
##  $ RichFactor    : num  0.0749 0.2593 0.062 0.1702 0.1091 ...
##  $ FoldEnrichment: num  2.86 9.89 2.36 6.49 4.16 ...
##  $ zScore        : num  5.65 7.59 4.94 6.19 5.47 ...
##  $ pvalue        : num  2.31e-06 4.49e-06 1.48e-05 2.62e-05 2.99e-05 ...
##  $ p.adjust      : num  0.00194 0.00194 0.00427 0.00517 0.00517 ...
##  $ qvalue        : num  0.00165 0.00165 0.00364 0.0044 0.0044 ...
##  $ geneID        : chr  "OPN3/PLPPR4/ADORA3/PTGER3/TACR1/SUCNR1/ACKR4/UTS2B/TRH/CXCL2/CXCL10/CXCL3/HTR1B/CHRM2/APLN/PENK/CHRM4/APLNR/HTR"| __truncated__ "MSMO1/HMGCS1/HMGCR/NSDHL/TM7SF2/IDI1/MVD" "OPN3/PLPPR4/ADORA3/PTGER3/TACR1/CALCRL/SUCNR1/ACKR4/UTS2B/TRH/CXCL2/CXCL10/CXCL3/HTR1B/WNT16/CALCR/CHRM2/APLN/P"| __truncated__ "PTGS2/IL1B/IL1A/IL12A/CXCL2/CXCL10/IL6/CCL2" ...
##  $ Count         : int  25 7 29 8 12 13 9 6 11 11 ...
## #...Citation
## Guangchuang Yu, Qing-Yu He. ReactomePA: an R/Bioconductor package for reactome pathway analysis and visualization. Molecular BioSystems. 2016, 12(2):477-479
```

```
slotNames(ePathDown) # Print out the slot names of the enrichment results.
```

```
##  [1] "result"        "pvalueCutoff"  "pAdjustMethod" "qvalueCutoff" 
##  [5] "organism"      "ontology"      "gene"          "keytype"      
##  [9] "universe"      "gene2Symbol"   "geneSets"      "readable"     
## [13] "termsim"       "method"        "dr"
```

```
ePathDownResults <- ePathDown@result # Extract the entire results from the result slot as a data frame. This include enriched and non-enriched terms.  

ePathDownEnriched <- as.data.frame(ePathDown) # Extract the enriched slot as a data frame. 

head(ePathDownEnriched, n = 2) # See the first two enriched pathways.
```

```
##                        ID                          Description GeneRatio
## R-HSA-373076 R-HSA-373076 Class A/1 (Rhodopsin-like receptors)    25/294
## R-HSA-191273 R-HSA-191273             Cholesterol biosynthesis     7/294
##                BgRatio RichFactor FoldEnrichment   zScore      pvalue
## R-HSA-373076 334/11214  0.0748503       2.855004 5.647119 2.31026e-06
## R-HSA-191273  27/11214  0.2592593       9.888889 7.587449 4.48546e-06
##                 p.adjust      qvalue
## R-HSA-373076 0.001937719 0.001647816
## R-HSA-191273 0.001937719 0.001647816
##                                                                                                                                                           geneID
## R-HSA-373076 OPN3/PLPPR4/ADORA3/PTGER3/TACR1/SUCNR1/ACKR4/UTS2B/TRH/CXCL2/CXCL10/CXCL3/HTR1B/CHRM2/APLN/PENK/CHRM4/APLNR/HTR7/PTGDR/PTGER2/CCL7/CCL2/MC4R/KISS1R
## R-HSA-191273                                                                                                            MSMO1/HMGCS1/HMGCR/NSDHL/TM7SF2/IDI1/MVD
##              Count
## R-HSA-373076    25
## R-HSA-191273     7
```

### Plot results of the enriched pathways

```
dotplot(ePathDown) + ggtitle("Dot plot for the top 10 enriched pathways") + theme(plot.title = element_text(hjust = 0.5, colour = "blue", face = "bold"), plot.title.position = "plot")
```

```
cnetplot(ePathDown, foldChange = sigDownGeneListEnt, color_category = "black", layout = igraph::layout_with_dh, color_edge = "category", node_label = "gene", size_edge = 0.8) + labs(title = "Term-gene network plot for the top 5 enriched pathways") + theme(plot.title = element_text(hjust = 0.5, colour = "blue", face = "bold"), plot.title.position = "plot")
```

```
cnetplot(ePathDown, showCategory = grep("lipid|stero", ePathDown$Description, value = T), color_edge = "category", node_label = "item", foldChange = sigDownGeneListEnt) + labs( title = "Term-gene network plot for the enriched pathways of lipogenesis") + theme(plot.title = element_text(hjust = 0.5, colour = "blue", face = "bold"), plot.title.position = "plot")
```

### Generate enrichment map for the enriched pathway

We need the term similarity matrix for generation of enrichment map,
but it is not generated during the enrichment analysis. The
*pairwise\_termsim()* function can generate the similarity matrix
and put it in the termsim slot.

```
ePathDownSimi <- pairwise_termsim(ePathDown) # This generates term similarity matrix and automatically adds the matrix to the S4 enrichment result in the termsim slot. 

# Scripts below generates the enrichment map for the top 25 enriched pathways. The default is 30 enriched terms. 
emapplot(ePathDownSimi, showCategory = 25) + labs( title = "Enrichment map for the top 25 enriched pathways") + theme(plot.title = element_text(hjust = 0.5, colour = "blue", face = "bold"), plot.title.position = "plot")
```

# FCS enrichment analysis for GO and KEGG pathways

## Prepare input gene list for FCS

```
geneList4FCS <- rawData$log2FoldChange

names(geneList4FCS) <- rawData$ENSEMBL

geneList4FCS <- sort(geneList4FCS, decreasing = T)

head(geneList4FCS)
```

```
## ENSG00000278041 ENSG00000088386 ENSG00000196090 ENSG00000251435 ENSG00000188460 
##        5.403378        5.306119        5.048141        5.031681        5.005066 
## ENSG00000152192 
##        4.897491
```

## Conduct enrichment analysis for GO terms using the *gseGO()* function, and overview the results.

```
gseBP <- gseGO(geneList = geneList4FCS, OrgDb = org.Hs.eg.db, keyType = "ENSEMBL", ont = "BP", pvalueCutoff = 0.1) # The original GSEA method suggestions of a pvalue threshold of 0.25. We use 0.1 here. 

gseBP
```

```
## #
## # Gene Set Enrichment Analysis
## #
## #...@organism     Homo sapiens 
## #...@setType      BP 
## #...@keytype      ENSEMBL 
## #...@geneList     Named num [1:35953] 5.4 5.31 5.05 5.03 5.01 ...
##  - attr(*, "names")= chr [1:35953] "ENSG00000278041" "ENSG00000088386" "ENSG00000196090" "ENSG00000251435" ...
## #...nPerm     
## #...pvalues adjusted by 'BH' with cutoff <0.1 
## #...25 enriched terms found
## 'data.frame':    25 obs. of  11 variables:
##  $ ID             : chr  "GO:0000070" "GO:0098813" "GO:0007059" "GO:0007094" ...
##  $ Description    : chr  "mitotic sister chromatid segregation" "nuclear chromosome segregation" "chromosome segregation" "mitotic spindle assembly checkpoint signaling" ...
##  $ setSize        : int  192 312 419 49 49 49 51 51 51 51 ...
##  $ enrichmentScore: num  0.416 0.366 0.342 0.582 0.582 ...
##  $ NES            : num  1.74 1.62 1.55 2 2 ...
##  $ pvalue         : num  1.76e-05 2.00e-05 1.62e-05 9.56e-05 9.56e-05 ...
##  $ p.adjust       : num  0.0406 0.0406 0.0406 0.0417 0.0417 ...
##  $ qvalue         : num  0.0405 0.0405 0.0405 0.0416 0.0416 ...
##  $ rank           : num  8291 11965 12838 8235 8235 ...
##  $ leading_edge   : chr  "tags=32%, list=23%, signal=25%" "tags=41%, list=33%, signal=28%" "tags=42%, list=36%, signal=27%" "tags=49%, list=23%, signal=38%" ...
##  $ core_enrichment: chr  "ENSG00000226650/ENSG00000125337/ENSG00000165828/ENSG00000161888/ENSG00000164695/ENSG00000186185/ENSG00000165480"| __truncated__ "ENSG00000054796/ENSG00000226650/ENSG00000198765/ENSG00000162039/ENSG00000125337/ENSG00000165828/ENSG00000161888"| __truncated__ "ENSG00000054796/ENSG00000226650/ENSG00000198765/ENSG00000162039/ENSG00000125337/ENSG00000165828/ENSG00000100162"| __truncated__ "ENSG00000165828/ENSG00000161888/ENSG00000165480/ENSG00000117724/ENSG00000154839/ENSG00000089685/ENSG00000166851"| __truncated__ ...
## #...Citation
## S Xu, E Hu, Y Cai, Z Xie, X Luo, L Zhan, W Tang, Q Wang, B Liu, R Wang, W Xie, T Wu, L Xie, G Yu. Using clusterProfiler to characterize multiomics data. Nature Protocols. 2024, 19(11):3292-3320
```

```
gseBP_results <- gseBP@result

head(gseBP_results, n = 1)
```

```
##                    ID                          Description setSize
## GO:0000070 GO:0000070 mitotic sister chromatid segregation     192
##            enrichmentScore      NES       pvalue   p.adjust     qvalue rank
## GO:0000070       0.4160622 1.744675 1.756162e-05 0.04061299 0.04052444 8291
##                              leading_edge
## GO:0000070 tags=32%, list=23%, signal=25%
##                                                                                                                                                                                                                                                                                                                                                                                                                                                                                                                                                                                                                                                                                                                                                                                                                                                                                                                                                                                                            core_enrichment
## GO:0000070 ENSG00000226650/ENSG00000125337/ENSG00000165828/ENSG00000161888/ENSG00000164695/ENSG00000186185/ENSG00000165480/ENSG00000117724/ENSG00000154839/ENSG00000167513/ENSG00000198901/ENSG00000118193/ENSG00000144031/ENSG00000102384/ENSG00000089685/ENSG00000166851/ENSG00000134690/ENSG00000178999/ENSG00000117399/ENSG00000088325/ENSG00000163808/ENSG00000117650/ENSG00000161800/ENSG00000071539/ENSG00000152253/ENSG00000237649/ENSG00000076382/ENSG00000121621/ENSG00000090889/ENSG00000101057/ENSG00000143228/ENSG00000137807/ENSG00000138160/ENSG00000010292/ENSG00000135476/ENSG00000108961/ENSG00000112742/ENSG00000175063/ENSG00000156970/ENSG00000164109/ENSG00000126787/ENSG00000142945/ENSG00000149503/ENSG00000122952/ENSG00000121152/ENSG00000170312/ENSG00000134057/ENSG00000137812/ENSG00000183765/ENSG00000169679/ENSG00000099812/ENSG00000109805/ENSG00000079616/ENSG00000276043/ENSG00000126215/ENSG00000138778/ENSG00000184445/ENSG00000146918/ENSG00000177602/ENSG00000136824/ENSG00000146670
```

## Visualization of the enriched gseGO results

```
 # Code below visualizes the activated and suppressed functions in one plot with two panels. 
dotplot(gseBP, label_format = 50, font.size = 11, split = ".sign", title = "Dot plot for enriched BP terms by GSEA") + facet_grid(.~.sign) + theme(plot.title = element_text(color = "blue", face = "bold", hjust = 0.5), plot.title.position = "plot")
```

```
gseBP <- pairwise_termsim(gseBP) # Generate the term similarity matrix.
# Generate the enrichment map.
emapplot(gseBP) + theme(plot.title = element_text(color = "blue", face = "bold", hjust = 0.5), plot.title.position = "plot") + ggtitle("Enrichment map plot for the enriched BP terms")
```

```
# Generate the term-gene network with ENSEMBL IDs. 
cnetplot(gseBP) + theme(plot.title = element_text(color = "blue", face = "bold", hjust = 0.5), plot.title.positon = "plot") + ggtitle("Term-gene network for enriched BP terms generated with default plotting settings")
```

## Generate term-gene network plot via *setReadable()* conversion of gene IDs.

The first script below converts *gseBP* to a new S4 object
with human readable gene symbols. The second script generates the
term-gene network plot with some customization using the ggplot2
grammar.

```
gseBP_readable <- setReadable(gseBP, OrgDb = org.Hs.eg.db, keyType = "ENSEMBL")

cnetplot(gseBP_readable, color_category = "black", color_edge = "category", layout = igraph::layout_with_dh) + ggtitle("Term-gene network plot with human readable gene labels") + theme(plot.title = element_text(hjust = 0.5, colour = "blue", face = "bold"), plot.title.position = "plot")
```

## Generate ridge plots

```
# The code below plots the ridgeplot with the default setting. As a result some terms names overlap with neighboring ones due to crowded terms (30 enriched terms plotted as default). 
ridgeplot(gseBP)
```

```
# The script below uses ggplot2 functions to additionally customize the plot (add x and y axis annotations, add figure title, and modify the text size for the Y axis labels)
ridgeplot(gseBP, label_format = 70) + labs(x = "Enrichment distribution", y = "Enriched terms") + ggtitle("gseBP ridgeplot") + theme(axis.text.y = element_text(size = 9)) + theme(plot.title = element_text(size = 14, colour = "blue", face = "bold"))
```

## Generate GSEA plot

The following code plots the GSEA plot for the first enriched term as
defined by *geneSetID = 1*. The figure title is defined by
*title = gseBP$Description[1]*.

```
gseaplot(gseBP, title = gseBP$Description[1], geneSetID = 1)
```

# FCS for KEGG pathway enrichment analysis

## Generate input gene list

```
geneList4KEGG <- rawDataEntrez$log2FoldChange # This generates a vector of log2FoldChange.

names(geneList4KEGG) <- rawDataEntrez$ENTREZID # This names each log2FoldChange value with its corresponding ENTREZID. 

geneList4KEGG <- sort(geneList4KEGG, decreasing = T) # This sorts the resulting vector from high to low based on log2FoldChange values. 

geneList4KEGGu <- geneList4KEGG[unique(names(geneList4KEGG))] # Since ENTREZID may have redundant during ID matching with ENSEMBL this steps just removes the redundatn ENTREZID using the unique() function. 

length(geneList4KEGG) # This checks the length of the gene list before removing the redundant ones.
```

```
## [1] 24111
```

```
length(geneList4KEGGu) # This checks the length of the gene list after removing the redundant ones.
```

```
## [1] 24071
```

```
head(geneList4KEGGu) # List the first six genes to review.
```

```
##     6564    11122     5457   347404   654346    55765 
## 5.306119 5.048141 4.897491 4.761269 4.687030 4.685198
```

## Construct S4 enrichment object and review the results

```
ePathKEGG <- gseKEGG(geneList4KEGGu, keyType = "ncbi-geneid", pvalueCutoff = 0.1) # Generate the enrichment S4 object using the gseKEGG() function. We use a higher p value since FCS is less sensitive. 

ePathKEGG # Print out overview of the results.
```

```
## #
## # Gene Set Enrichment Analysis
## #
## #...@organism     hsa 
## #...@setType      KEGG 
## #...@keytype      ncbi-geneid 
## #...@geneList     Named num [1:24071] 5.31 5.05 4.9 4.76 4.69 ...
##  - attr(*, "names")= chr [1:24071] "6564" "11122" "5457" "347404" ...
## #...nPerm     
## #...pvalues adjusted by 'BH' with cutoff <0.1 
## #...19 enriched terms found
## 'data.frame':    19 obs. of  11 variables:
##  $ ID             : chr  "hsa05323" "hsa04657" "hsa05322" "hsa04060" ...
##  $ Description    : chr  "Rheumatoid arthritis" "IL-17 signaling pathway" "Systemic lupus erythematosus" "Cytokine-cytokine receptor interaction" ...
##  $ setSize        : int  81 86 114 227 54 78 118 193 137 44 ...
##  $ enrichmentScore: num  -0.538 -0.519 -0.481 -0.386 -0.56 ...
##  $ NES            : num  -1.94 -1.91 -1.83 -1.61 -1.9 ...
##  $ pvalue         : num  7.37e-06 1.90e-05 1.50e-05 5.01e-05 7.94e-05 ...
##  $ p.adjust       : num  0.00222 0.00222 0.00222 0.0044 0.00557 ...
##  $ qvalue         : num  0.00201 0.00201 0.00201 0.00397 0.00503 ...
##  $ rank           : num  6911 3487 5495 4832 5850 ...
##  $ leading_edge   : chr  "tags=60%, list=29%, signal=43%" "tags=28%, list=14%, signal=24%" "tags=54%, list=23%, signal=41%" "tags=38%, list=20%, signal=31%" ...
##  $ core_enrichment: chr  "537/9114/9550/2919/10312/535/3108/528/9296/3112/8992/523/526/3383/245973/3123/6387/10673/7124/1437/3683/1493/94"| __truncated__ "6361/5596/4318/6374/7128/4312/6372/2920/3569/2921/3567/3576/6347/2353/3553/4322/4314/6364/6356/3627/5743/2354/6354/27189" "8353/717/3021/716/3586/3017/8330/92815/3123/7124/8349/6741/128312/85236/8345/333932/731/941/8342/286436/8364/89"| __truncated__ "920/6387/50604/133396/10673/3554/7124/3570/3953/8200/27178/1437/3577/58985/6846/1232/1524/10563/944/3559/6361/6"| __truncated__ ...
## #...Citation
## S Xu, E Hu, Y Cai, Z Xie, X Luo, L Zhan, W Tang, Q Wang, B Liu, R Wang, W Xie, T Wu, L Xie, G Yu. Using clusterProfiler to characterize multiomics data. Nature Protocols. 2024, 19(11):3292-3320
```

```
slotNames(ePathKEGG) # Print out the slot names.
```

```
##  [1] "result"      "organism"    "setType"     "geneSets"    "geneList"   
##  [6] "keytype"     "permScores"  "params"      "gene2Symbol" "readable"   
## [11] "termsim"     "method"      "dr"
```

```
ePathKEGG_results <- ePathKEGG@result  # Generate the result data frame. 

ePathKEGG_results[1,] # Review the first enriched pathway.
```

```
##                ID          Description setSize enrichmentScore       NES
## hsa05323 hsa05323 Rheumatoid arthritis      81      -0.5381607 -1.942503
##                pvalue    p.adjust      qvalue rank
## hsa05323 7.371495e-06 0.002224415 0.002007944 6911
##                            leading_edge
## hsa05323 tags=60%, list=29%, signal=43%
##                                                                                                                                                                                                                                           core_enrichment
## hsa05323 537/9114/9550/2919/10312/535/3108/528/9296/3112/8992/523/526/3383/245973/3123/6387/10673/7124/1437/3683/1493/941/6374/1514/527/3589/3119/4312/6372/7042/2920/3569/2921/284/6349/3576/3111/6347/940/2353/3553/3122/4314/6364/3552/50617/3120/3109
```

## visualization of the enriched KEGG pathways

### dotplot

```
# This script below splits the dot plots based on the sign column, which is internally prepared, into two panels arranged in a single row. The facet_grid() function has a "row ~ column" format. 
dotplot(ePathKEGG, title = "Split dot plots for KEGG pathway enrichment", split = ".sign") + facet_grid(".~.sign") + theme(axis.text.y = element_text(size = 10, face = "bold")) + ggtitle("Dot plot for the enriched KEGG pathways with separate panels for activated and suppressed pathways") + theme(plot.title = element_text(color = "blue", hjust = 0.5, face = "bold", size = 10), plot.title.position = "plot")
```

### Ridgeplot

```
ridgeplot(ePathKEGG) + theme(axis.text.y = element_text(size = 10)) + ggtitle("Ridge plot for the enriched KEGG pathways for activated and suppressed pathways") + theme(plot.title = element_text(color = "blue", hjust = 0.5, face = "bold"), plot.title.position = "plot")
```

### gseaplot.

The plot title is the term name, which is generated automatically
with a code.

```
# The script below generates the GSEA plot for the second enriched KEGG pathway with the term name as the figure title. 
gseaplot(ePathKEGG, title = ePathKEGG@result$Description[2], geneSetID = 2)
```

### Visualize the enriched KEGG pathways with term-gene network

Generate cnetplot with the default settings. The network plot uses
ENTREZID as gene labels.

```
cnetplot(ePathKEGG) + ggtitle("Term-gene network plot for the enriched KEGG pathways with ENTREZID as gene labels") + theme(plot.title = element_text(color = "blue", hjust = 1, face = "bold", size = 10), plot.title.position = "plot")
```

Convert the enrichment result S4 object to one with human readable
gene labels.

```
ePathKEGGreadable <- setReadable(ePathKEGG, OrgDb = org.Hs.eg.db, keyType = "ENTREZID")

ePathKEGG@result$core_enrichment[1] # Print out the gene list in the leading edge for the top-1 enriched KEGG pathway.
```

```
## [1] "537/9114/9550/2919/10312/535/3108/528/9296/3112/8992/523/526/3383/245973/3123/6387/10673/7124/1437/3683/1493/941/6374/1514/527/3589/3119/4312/6372/7042/2920/3569/2921/284/6349/3576/3111/6347/940/2353/3553/3122/4314/6364/3552/50617/3120/3109"
```

```
ePathKEGG$core_enrichment[1] # This simple code gives the same results as the code above since you can treat the S4 enrichment result object as a data frame to process.
```

```
## [1] "537/9114/9550/2919/10312/535/3108/528/9296/3112/8992/523/526/3383/245973/3123/6387/10673/7124/1437/3683/1493/941/6374/1514/527/3589/3119/4312/6372/7042/2920/3569/2921/284/6349/3576/3111/6347/940/2353/3553/3122/4314/6364/3552/50617/3120/3109"
```

```
identical(ePathKEGG@result$core_enrichment[1], ePathKEGG$core_enrichment[1]) # This code proves that the above two scripts produce the same result.
```

```
## [1] TRUE
```

```
ePathKEGGreadable@result$core_enrichment[1]
```

```
## [1] "ATP6AP1/ATP6V0D1/ATP6V1G1/CXCL1/TCIRG1/ATP6V0A1/HLA-DMA/ATP6V1C1/ATP6V1F/HLA-DOB/ATP6V0E1/ATP6V1A/ATP6V1B2/ICAM1/ATP6V1C2/HLA-DRB1/CXCL12/TNFSF13B/TNF/CSF2/ITGAL/CTLA4/CD80/CXCL5/CTSL/ATP6V0C/IL11/HLA-DQB1/MMP1/CXCL6/TGFB2/CXCL2/IL6/CXCL3/ANGPT1/CCL3L1/CXCL8/HLA-DOA/CCL2/CD28/FOS/IL1B/HLA-DRA/MMP3/CCL20/IL1A/ATP6V0A4/HLA-DQB2/HLA-DMB"
```

Generate and customize the term-gene network with human readable gene
labels. We use ggplot2 grammar to customize the plot. ggplot2 functions
are added with a + sign as shown below.

```
cnetplot(ePathKEGGreadable, showCategory = 3, color_category = "black", color_edge = "category", foldChange = geneList4KEGGu) + ggtitle("Term-gene network plot for enriched KEGG pathways with human readable gene labels") + theme(plot.title = element_text(hjust = 1, face = "bold", colour = "blue", size = 11), plot.title.position = "plot")
```
